# High-Performance Perovskite Solar Cells and Modules via Slot-Die Coating: Engineering Advances, Photoluminescence Insights, and Future Perspectives

**DOI:** 10.1007/s40820-026-02134-7

**Published:** 2026-03-28

**Authors:** Li’an Peng, Yonglong Mao, Yuxia Yin, Yuxin Chen, Teng Zhang, Shengye Jin, Jun Zhang

**Affiliations:** 1 Shandong Key Laboratory of Intelligent Energy Materials, School of Materials Science and Engineering, China University of Petroleum (East China), Qingdao, 266580 People’s Republic of China; 2 State Key Laboratory of Molecular Reaction Dynamics, Dalian Institute of Chemical Physics, Chinese Academy of Sciences, Dalian, 116023 People’s Republic of China

**Keywords:** Slot-die coating, Perovskite photovoltaics, Process–structure–property relationship, Photoluminescence, Laboratory-to-fab transition

## Abstract

Slot-die coating has solidified its role as the preeminent industrial-scale deposition technique for perovskite photovoltaics, combining exceptional material utilization, direct compatibility with roll-to-roll production, and high-throughput capability. This review comprehensively surveys recent advances in slot-die-coated perovskite solar cells and modules, with a focused analysis on the governing principles behind the structure–property–performance relationship. We first deconstruct how coating methodologies—including one-step, two-step, and hybrid processes—dictate film morphology and crystalline quality. We then elaborate on perovskite ink engineering, emphasizing the critical role played by solvent selection and functional additives in controlling nucleation kinetics and crystal growth during large-area deposition. Furthermore, we examine innovative interface modulation strategies that enhance film integrity, suppress defects, and mitigate ion migration. A key distinctive feature of this review is its emphasis on advanced photoluminescence (PL) characterization techniques—such as in situ PL, PL imaging, and time-resolved PL—which provide unparalleled insights into crystallization pathways, defect distribution, and charge carrier dynamics. These tools are indispensable for mechanistic decoding and rational optimization of the slot-die process. Finally, we outline pressing research directions, highlighting the necessity to overcome persistent challenges in operational stability, efficiency–stability trade-offs, the establishment of unified stability assessment protocols, and manufacturing reproducibility to ultimately bridge the laboratory-to-fab gap and accelerate the commercialization of slot-die-coated perovskite solar modules.

## Highlights

- Slot-die coating is critically assessed as the pivotal industrial deposition technique for perovskite photovoltaics, supported by its proven capability to produce high-efficiency, large-area modules.
- Advanced photoluminescence techniques unlock a paradigm shift, transforming slot-die process optimization from empirical tuning to rational design via direct visualization of crystallization and defect dynamics.
- A holistic roadmap is outlined to bridge the laboratory-to-fab gap, integrating crystallization control, green manufacturing, intrinsic stabilization, standardized assessment, and smart, data-driven production.

## Graphical abstract

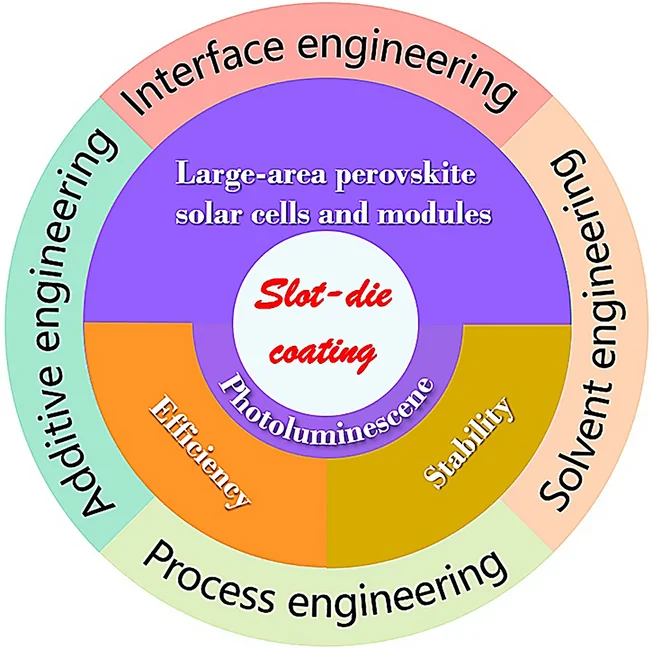

## Introduction

Perovskite solar cells (PSCs) have emerged as a transformative force in photovoltaics, boasting unparalleled optoelectronic properties such as a high absorption coefficient, tunable direct bandgap, and exceptionally long charge carrier diffusion lengths [1–3]. These merits have propelled laboratory-scale devices, predominantly fabricated via spin coating, to certified power conversion efficiencies (PCEs) exceeding 27%, rivaling established technologies [4, 5]. However, this laboratory feast is now facing the stern test of industrialization: the transition from small-area champions to large-area modules unveils a critical efficiency–stability–uniformity trilemma. The PCEs of most scaled-up perovskite solar modules (PSMs) linger below 20%, primarily plagued by insufficient morphological and optoelectronic uniformity over large areas [6–9]. Critically, this non-uniformity is not merely a culprit for efficiency loss but also the root cause of localized degradation hot spots, severely undermining operational stability under real-world thermal, electrical, and environmental stresses [10–12]. Therefore, to cross the “valley of death” from laboratory to fab, a paradigm shift from stochastic processes to controllable manufacturing is urgently required.

The inherent limitations of spin coating—its stochastic crystallization dynamics, low material yield, and poor scalability—have catalyzed the exploration of alternative printing and coating methods [13–15], including spray coating [16, 17], inkjet printing [18, 19], D-bar coating [20, 21], blade coating [22, 23], maker pen writing [24], and slot-die coating [25, 26]. Among these, slot-die coating stands out as the most promising and versatile technique for gigawatt-scale manufacturing, particularly due to its quintessential compatibility with roll-to-roll (R2R) production lines. Its defining advantages include: (i) exceptional film uniformity and thickness control over large areas, enabled by precise metering of the ink via an enclosed fluid delivery system; (ii) near-100% material utilization, drastically reducing waste and cost; (iii) non-contact coating that minimizes substrate damage and contamination; (iv) inherent scalability and high throughput, seamlessly integrating with upstream and downstream processes; and (v) the ability to coat multilayer stacks (e.g., charge transport layers, perovskite, electrodes) in a sequential, in-line manner. Crucially, slot-die coating is not merely a tool substitution but represents a fundamental shift in the physicochemical landscape of film formation. The transition from centrifugal forces to controlled meniscus dynamics, coupled with distinct evaporation pathways and macroscopic nucleation/growth kinetics, introduces unique challenges and opportunities that directly dictate film quality, defect proliferation, and ultimate module performance and reliability. This shift necessitates a parallel transformation in material design philosophy: from formulations optimized for the extreme, non-equilibrium conditions of spin coating, to “coating-oriented inks” engineered for the evaporation-controlled, shear-dominated regime of slot-die processes. Encouragingly, the remarkable progress in this field is powerfully evidenced by a recent milestone: a slot-die-coated PSM with a substantial aperture area of 715.10 cm^2^ has achieved a high PCE of 22.80% [27]. This achievement not only narrows the efficiency gap with spin-coated counterparts but, more importantly, demonstrates exceptional uniformity, offering a compelling solution to the uniformity–stability nexus and robustly validating the formidable commercial potential of slot-die coating.

In this context, this review aims to systematically decipher how slot-die coating technology can solve the core challenges of perovskite industrialization. We begin with a general description of the slot-die coating process. Next, advancements in PCEs of slot-die-coated PSCs/PSMs are summarized in detail. We emphasize that scientific optimization of processing parameters (e.g., one-step and two-step) and post-deposition treatments (e.g., gas quenching, vacuum flashing, anti-solvent extraction) constitute the cornerstone for enhancing the morphological uniformity of perovskite films. Precise ink modulation via solvent engineering and additive engineering dictates nucleation/growth dynamics. Considering that interfacial recombination and ion migration remain the primary limiting factors affecting device performance, this review systematically discusses recent advances in interface engineering strategies. A pivotal highlight and unique value of this review lies in the integration of non-contact, high-throughput photoluminescence (PL) characterization techniques—including in situ PL, PL imaging, and time-resolved PL—as indispensable diagnostic tools. These methods provide unparalleled, mechanistic insights into crystallization progression, defect distribution, and charge carrier dynamics across large areas, offering critical feedback for rational process optimization. Finally, building upon these foundations, we will prospect a future research and development (R&D) roadmap to address lingering challenges in efficiency, stability, and manufacturability, charting a course toward the commercial reality of slot-die-coated PSMs.

## Fundamentals and Principles of Slot-Die Coating

### Process Configuration and Fluid Mechanics

**Fig. 1:**
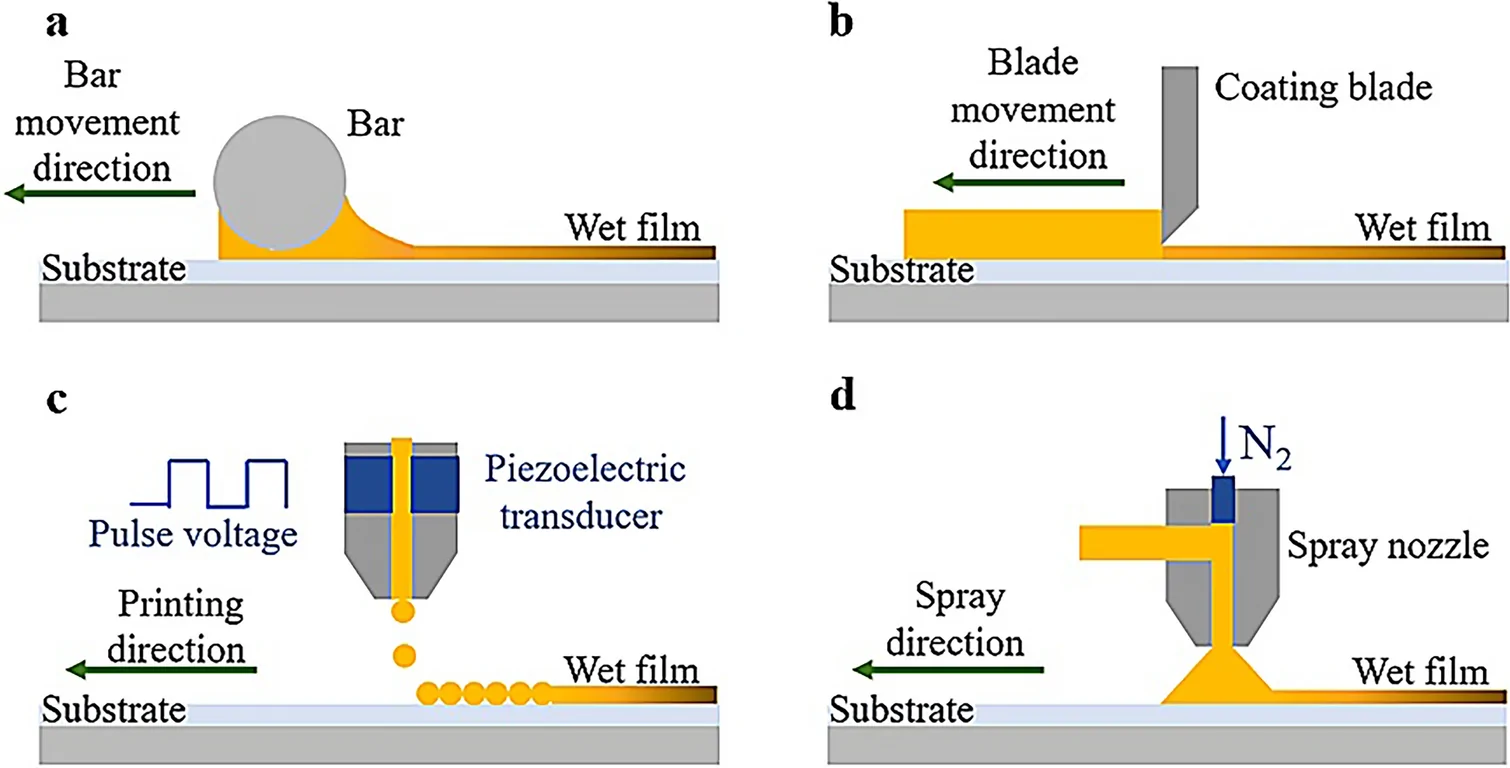
Schematic illustrations of four scalable coating techniques: **a** D-bar coating, **b** blade coating, **c** inkjet printing, and **d** spray coating

With continuous technological advancements, slot-die coating has become a widely used thin-film deposition method in optoelectronics and semiconductors packaging [28, 29]. Specifically, the slot-die coating offers exceptional material usage, precise film thickness control, and excellent compatibility with the R2R process [30, 31]. These attributes render it more advantageous over other scaled-up perovskite film deposition methods such as D-bar coating, blade coating, inkjet printing, and spray coating (Fig. 1a–d).

**Fig. 2:**
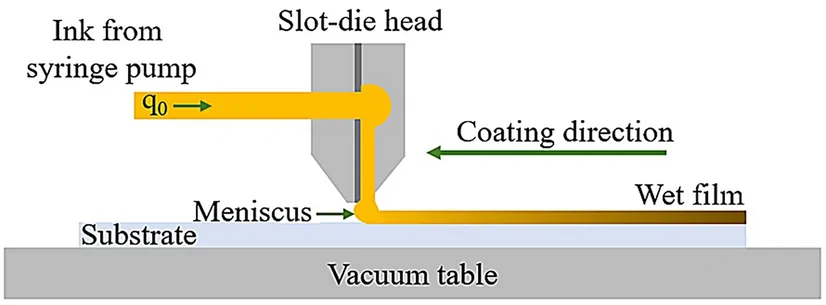
Schematic illustration of the slot-die coating process

In a typical slot-die coating configuration, the coating head is positioned in close proximity to the substrate. The ink is delivered to the coating head via a programmed syringe pump (Fig. 2). During the coating process, upstream and downstream ink menisci are formed between the coating head and substrate, facilitating the deposition of the homogeneous wet films as the substrate traverses the coating head (or the reverse). Compared with blade coating, an automated liquid injecting system has been integrated into the slot-die coating, which guarantees the continuous supply of the coating inks [32–34]. This is critical as the fluid dynamics of the coating inks directly influence the quality and uniformity of the wet films.

Following the fluid mechanics, the low-flow limit (*C*_*low-flow*_) represents the minimum flow condition required to ensure the continuity of the slot-die-coated films. According to Eq. (2), the *C*_*low-flow*_ is determined by the distance between the coating head and the substrate (*h*) and the pre-seated wet film thickness (*t*) [35]. During a specific coating process, the capillary number (*Ca*) should be lower than or equal to the *C*_*low-flow*_ to guarantee the complete coverage of the wet films [36]. Therefore, the viscosity of the ink (*µ*), the moving speed of the coating head (*ν*), and the surface tension of the downstream meniscus (*σ*) should be precisely adjusted (Eq. 1).

1 $$C = \frac{\mu \nu }{\sigma }$$

2 $$C_{{low - flow}} = {\text{ }}0.65~\left( {\frac{2}{{\frac{H}{t} - 1}}} \right)^{{\frac{3}{2}}}$$

Where *Ca* is the capillary number, *µ* is the ink viscosity; *ν* is the substrate moving speed, *σ* is the surface tension of the downstream meniscus, *H* is the gap between substrate and coating head, and *t* is the thickness of the wet films.

Despite advances in slot-die coating, a persistent knowledge gap exists in correlating coating parameters (speed, flow rate, gap, solvent removal rate) with perovskite film quality. The process exhibits high sensitivity to ambient conditions (e.g., temperature fluctuations exceeding 5 °C, relative humidity (RH) > 60%), critically affecting final properties [37, 38]. To optimize drying kinetics, auxiliary techniques—in situ annealing [39, 40], N_2_ knife blowing [40, 41], anti-solvent quenching [42, 43], and vacuum-assisted flashing [44, 45]—are integrated with slot-die coating, speeding up the drying kinetics and enabling homogeneous pinhole-free films with nanometer surface roughness. However, fundamental understanding of synergistic/competitive drying kinetics (e.g., vapor pressure incompatibility between N_2_ knife and vacuum flashing) among these hybrid approaches remains imperative.

### Rheological Fundamentals and the Coating Operating Window

While the basic fluid mechanics of slot-die coating are governed by Eqs. (1) and (2), the successful translation to high-speed, R2R production of perovskite films demands a deeper understanding of the rheological properties of the precursor ink. Unlike Newtonian fluids, perovskite precursor solutions are typically complex colloidal dispersions containing lead halide complexes, organic salts, and molecular adducts, exhibiting non-ideal rheology that critically impacts coating stability and film uniformity [46, 47].

#### Key Rheological Behaviors in Perovskite Inks

Shear Thinning and Thixotropy: Most perovskite inks exhibit shear-thinning behavior, where viscosity (µ) decreases under applied shear stress (e.g., during pumping and flow through the slot-die head) [48, 49]. This is beneficial for reducing flow resistance during high-speed coating. However, a related property, thixotropy [50, 51], which refers to the time-dependent recovery of viscosity after shear is removed, is of paramount importance. In R2R processes, the ink experiences high shear in the die, but once deposited onto the moving substrate, shear rapidly drops to near zero. If the ink’s microstructure (e.g., colloidal clusters, adduct networks) recovers too slowly (low thixotropy), the wet film may remain fluid for too long, leading to meniscus instability and uneven film thickness. Conversely, if recovery is too rapid (high thixotropy), the ink may gel prematurely at the die lips, causing ribbing (parallel stripes perpendicular to coating direction) or even clogging. Optimal thixotropy ensures the ink flows smoothly during deposition but quickly gains enough viscosity post-deposition to “freeze” the coated morphology before defects can form.

Viscoelasticity: The elastic component of the ink (its ability to store energy under deformation) influences the meniscus shape and stability between the die and substrate. Excessive elasticity can lead to meniscus draw-up or fingering instabilities, breaking the uniform wet film line. For perovskite inks, viscoelasticity often arises from long-chain polymers or entangled colloidal structures formed by solvents and additives.

#### Correlation with Common Coating Defects

The non-Newtonian rheology of perovskite inks directly manifests in specific coating defects if not properly managed:

Ribbing: Primarily driven by an imbalance between capillary pressure and viscous forces. It occurs when the capillary number (Ca) exceeds a critical value for a given geometry (gap-to-thickness ratio, H/t). In practice, for shear-thinning inks, the effective viscosity at the high shear rate in the coating bead is lower, which can inadvertently push the process into a ribbing-prone regime if speed (ν) is not carefully balanced with flow rate (Q). Ribbing is often a sign of operating outside the stable coating window. Meniscus Instability and Dewetting: This is linked to ink surface tension (σ), substrate wettability, and the ink’s viscosity recovery rate (thixotropy) after deposition. If the ink’s surface tension is too high or its viscosity too low upon deposition, it may retract from the substrate, causing pinholes or bare spots. Thixotropic recovery helps “pin” the contact line and prevent dewetting.

Meniscus Instability and Dewetting: This is linked to ink surface tension (σ), substrate wettability, and the ink’s viscosity recovery rate (thixotropy) after deposition. If the ink’s surface tension is too high or its viscosity too low upon deposition, it may retract from the substrate, causing pinholes or bare spots. Thixotropic recovery helps “pin” the contact line and prevent dewetting [52, 53].

#### Defining the “Operating Window” for Slot-Die Coating

**Fig. 3:**
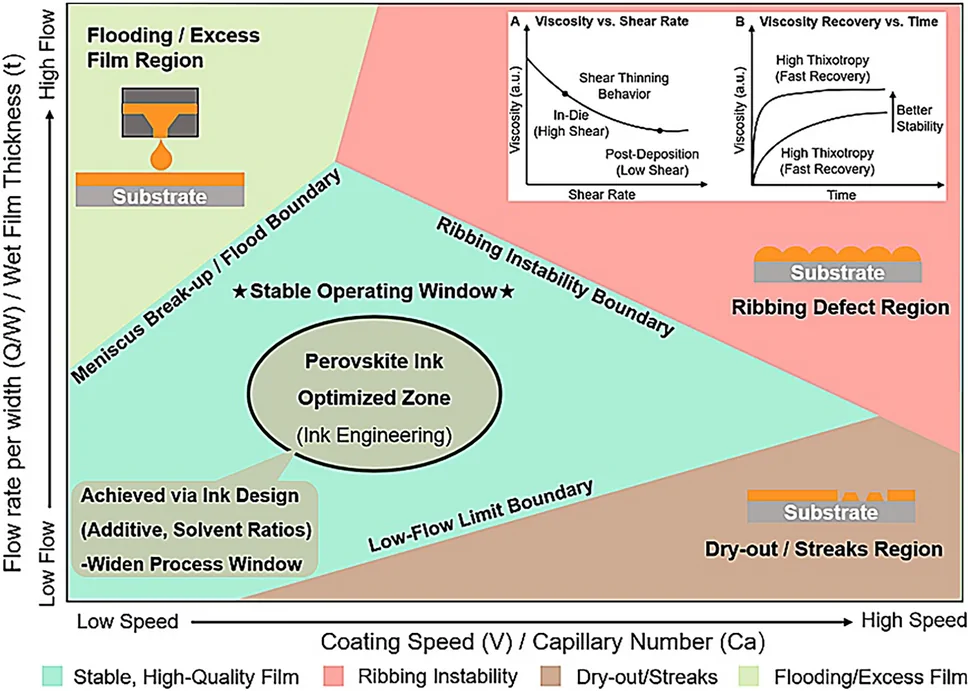
Conceptual operating window for slot-die coating of perovskite inks

The successful operation of slot-die coating is confined to a multidimensional operating window, defined by key process parameters and ink properties. Figure 3 illustrates a typical operating window. The low-flow limit (*C*_*low-flow*_) boundary (Eq. 2) defines the minimum flow rate to maintain a continuous film. The ribbing instability boundary marks the onset of periodic thickness variations. The meniscus breakup or dry-out boundary occurs at high speeds/low flow rates where the ink supply cannot keep up with substrate motion. The ideal processing zone is a region within these boundaries where stable, uniform films are produced. For perovskite inks, this window is often narrow and shifts significantly with ink formulation (solvent ratio, additive concentration, colloidal content) and ambient conditions [54–56].

#### Implications for R2R Manufacturing and Process Design

In a high-speed R2R line, the coating speed is fixed by the web tension and drive system. Therefore, achieving a defect-free film requires precise synchronization of ink rheology with process parameters. The ink must be formulated to have the right shear-thinning profile to flow at the line speed and the appropriate thixotropic recovery to stabilize the wet film before entering the drying zone. Understanding and characterizing these rheological properties (via rheometry measuring viscosity vs. shear rate, and recovery loops) is not merely academic but a critical step in process design and scale-up, bridging the gap between laboratory-scale optimization and gigawatt-scale production [57, 58].

## Performance Evolution and Stability Assessment of Slot-Die-Coated PSCs and PSMs

### Overview and Performance Evolution

**Fig. 4:**
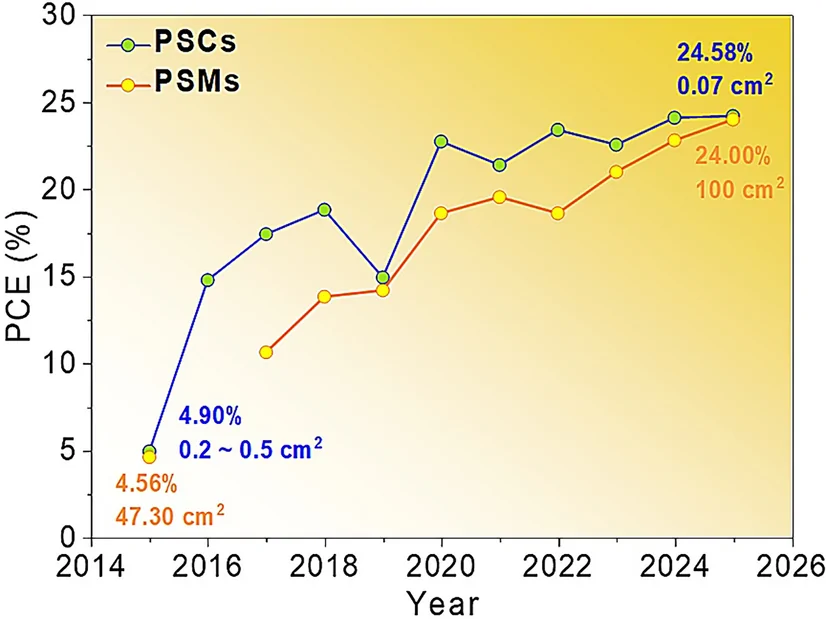
Evolution of PCEs for slot-die-coated PSCs and PSMs from 2015 to 2025

In 2015, Vak et al*.* pioneered the application of slot-die coating technology to PSCs, where a PCE exceeding 10% conclusively demonstrated the viability of this approach, heralding scalable perovskite photovoltaics [59]. Subsequently, significant research efforts have been devoted to slot-die-coated PSCs/PSMs. Figure 4 illustrates the evolution of PCEs for these devices in photovoltaic technology from 2015 to 2025, with detailed performance values summarized in Tables 1 and 2. To standardize performance comparisons, the Green module criteria [60, 61]—which define a photovoltaic module as having an active area ≥ 10 cm^2^—are applied to distinguish cells from modules throughout this review.

**Table 1:** Cell configurations and *J–V* scan photovoltaic parameters of the slot-die-coated PSCs

Year	Device configuration	Active area (cm^2^)	*Jsc* (mA cm^2^)/*I* (mA)	*Voc* (V)	*FF* (%)	*Eff* (%)	Refs
2015	ITO/PEDOT:PSS/MAPbI_3-x_Cl_x_/PCBM/ZnO/Ag	0.2 ~ 0.5	10.90	0.90	50.00	4.90	[64]
2015	ITO/PEDOT:PSS/C3-SAM/MAPbI_3-x_Cl_x_/PC_61_BM/ZnO/Ag	0.55	13.70	0.98	38.00	5.10	[65]
2015	ITO/ZnO/MAPbI_3_/doped P3HT/Ag	0.1	19.35	0.96	63.00	11.60	[59]
2015	ITO/ZnO/MAPbI_3_/P3HT/Ag	0.1	20.38	0.98	59.88	11.96	[66]
2016	FTO/TiO_2_/MAPbI_3_/Spiro-OMeTAD/Au	0.0625	16.80	0.73	75.60	9.20	[67]
2016	ITO/ZnO/MAPbI_3_/Bifluo-OMeTAD/MoO_x_/Ag	0.1	19.68	1.10	68.11	14.75	[68]
2017	PET/ITO/PEDOT:PSS/MAPbI_3-x_Cl_x_/PCBM/Ag	0.12	10.52	0.75	37.77	2.91	[69]
2017	PET/ITO/ZnO/MAPbI_3_/P3HT/Au	1.0	–	–	–	3.60	[70]
2017	ITO/FrGO/MAPbI_3_/PC_61_BM/BCP/Ag	0.1	16.34	1.00	76.00	12.52	[71]
2017	ITO/ZnO/MAPbI_3_/Bifluo-OMeTAD/MoO_3_/Ag	0.1	21.30	1.08	62.30	14.40	[72]
2017	FTO/ZnO/MAPbI_3_/Carbon	1.0	22.90	1.05	63.00	15.10	[45]
2017	ITO/TiO_2_/MAPbI_3-x_Cl_x_/Spiro-OMeTAD/Au	0.16	22.40	1.044	76.00	17.40	[73]
2018	PET/ITO/PEDOT:PSS/MAPbI_3_/PCBM/Ag	0.1	20.50	0.92	35.00	6.50	[74]
2018	FTO/c-TiO_2_/s-TiO_2_/MAPbI_3_/Spiro-OMeTAD/Au	0.09	16.70	0.96	67.20	11.00	[75]
2018	ITO/PEDOT:PSS/MAPbI_3_/PCBM/PEI/Ag	0.3	16.64	0.95	72.40	11.40	[76]
2018	FTO/c-TiO_2_/m-TiO_2_/MAPbI_3-x_Cl_x_/Spiro-OMeTAD/Au	0.09	18.33	0.89	72.68	11.74	[77]
2018	ITO/ZnO NPs/MAPbl_3_/Bifluo-OMeTAD/MoO_3_/Ag	0.1	17.21	1.10	67.25	12.73	[41]
2018	ITO/PEDOT:PSS/MAPbI_3_/C_60_/PCBM/BCP/Ag	0.1	19.90	0.89	74.00	13.30	[78]
2018	PET/ITO/SnO_2_/Cs_0.15_FA_0.85_PbI_2.85_Br_0.15_/Spiro-OMeTAD/Au	0.09	20.70	1.029	71.50	15.20	[79]
2018	ITO/PEDOT:PSS/MAPbI_3_/PCBM/Ca/Al	0.1	19.79	1.02	77.15	15.57	[80]
2018	ITO/SnO_2_/MAPbI_3_/Spiro-OMeTAD/Au	0.06	21.50	1.10	76.00	18.00	[81]
2018	FTO/SnO_2_ NPs/MAPbI_3_/Spiro-OMeTAD/Au	0.096	22.00	1.12	76.30	18.80	[82]
2019	PET/ITO/PEDOT:PSS/(BA)_2_(MA)_3_Pb_4_I_13_/PC_61_BM/PEIE/Ag	0.1	13.60	1.02	57.8	8.00	[83]
2019	PEN/ITO/SnO_2_/FAMACs-perovskite/Spiro-OMeTAD/Ag	0.07	16.82	0.98	63.99	10.57	[84]
2019	PET/ITO/PEDOT:PSS/MAPbI_3_/PCBM/BCP/Ag	0.09	18.50	0.98	72.00	12.80	[85]
2019	ITO/PEDOT:PSS/MA_0.6_FA_0.38_Cs_0.02_PbI_2.975_Br_0.025_/PCBM/PEIE/Ag	0.1	19.43	1.06	72.40	14.91	[86]
2020	PET/ITO/PEDOT:PSS/quasi-2D/3D perovskite/PC_61_BM/PEIE/Ag	0.1	13.60	1.04	66.00	9.30	[87]
2020	FTO/NiO_x_/MAPbI_3_/PCBM/PEI/Ag	3.78	2.85	5.73	63.34	10.34	[88]
2020	FTO/NiO_x_/MAPbI_3_/PCBM/PEI/Ag	0.09	18.73	1.02	74.91	14.30	[88]
2020	ITO/PTAA/MAPbI_3_(Cys.HCl)/C_60_/BCP/Cu	1.7	20.53	4.05	69.09	14.43	[89]
2020	FTO/TiO_2_/MAPbI_3_/Spiro-OMeTAD/Au	0.125	20.98	1.046	66.10	14.51	[39]
2020	ITO/SnO_2_/CsFAMA-perovskite/Spiro-OMeTAD/Ag	0.07	20.70	1.06	65.90	14.55	[90]
2020	ITO/PTAA/MAPbI_3_(Cys.HCl)/C_60_/BCP/Cu	0.1	24.79	1.096	80.29	21.83	[89]
2020	FTO/TiO_2_/FA_0.91_Cs_0.09_PbI_3_/PC_61_BM)/Ag	0.1	25.60	1.125	78.90	22.73	[91]
2021	PET/ITO/Poly-TPD/MAPbI_3_/PCBM/Au	0.16	10.27	0.96	34.00	3.30	[92]
2021	PET/ITO/PEDOT:PSS/MA_0.61_FA_0.37_Cs_0.02_PbI_2.88_Br_0.12_/PCBM/PEIE/Au	0.1	20.06	0.94	59.14	11.15	[93]
2021	PET/ITO/SnO_2_/Cs_0.1_(MA_0.15_FA_0.85_)_0.9_Pb(I_0.85_Br_0.15_)_3_/Spiro-OMeTAD/MoO_3_/Ag	0.12	19.81	0.95	70.00	13.41	[42]
2021	ITO/2PACz/MAPbI_3_/C_60_/BCP/Cu	2.2	14.38	3.189	69.91	14.57	[94]
2021	FTO/c-TiO_2_/m-TiO_2_/Cs_0.17_FA_0.83_Pb(I_0.83_Br_0.17_)_3_/Spiro-OMeTAD/Au	0.09	20.30	1.14	75.00	17.50	[95]
2021	FTO/SnO_2_/Cs_0.16_FA_0.84_Pb(I_0.88_Br_0.12_)_3_/Spiro-OMeTAD/Au	0.09	22.40	1.024	78.60	18.00	[40]
2021	FTO/c-TiO_2_/m-TiO_2_/GO-K/Cs_0.1_FA_0.9_Pb(I_0.94_Br_0.06_)_3_/Spiro-OMeTAD/Au	0.09	23.28	1.04	77.66	18.30	[96]
2021	FTO/NiMgLiO/FA_0.83_Cs_0.17_PbI_2.83_Br_0.17_/LiF/C_60_/BCP/Bi/Ag	1.0	21.8	1.08	78.90	18.50	[43]
2021	FTO/c-TiO_2_/SnO_2_/Cs_0.05_MA_0.4_FA_0.55_Pb(I_0.96_Br_0.04_)_3_/Spiro-OMeTAD/Au	\	22.5	1.09	77.10	19.00	[97]
2021	ITO/2PACz/MAPbI_3_/C_60_/BCP/Cu	0.16	23.67	1.138	77.00	20.83	[94]
2021	ITO/PTAA/KSCN-MAPbI_3_/C_60_/BCP/Ag	0.089	24.54	1.113	78.26	21.38	[98]
2022	ITO/PEDOT:PSS/Cs_0.07_FA_0.79_MA_0.14_Pb(I_0.83_Br_0.17_)_3_/PCBM/PEIE/Au	0.2	21.57	0.94	58.96	11.96	[99]
2022	ITO/NiO_x_/Cs_0.175_FA_0.750_MA_0.075_Pb(I_0.880_Br_0.120_)_3_/PCBM/ZnO/Ag	\	22.84	1.04	61.57	14.63	[100]
2022	ITO/NiO_x_/CsFAPbI_3-x_Cl_x_/PCBM/BCP/Cu	2.1	21.74	2.03	68.00	14.90	[101]
2022	ITO/NiO_x_-NPs/Cs_0.175_FA_0.750_MA_0.075_Pb(I_0.880_Br_0.120_)_3_/PCBM/ZnO NPs/Ag	0.1	22.03	1.05	66.74	15.40	[102]
2022	ITO/NiO_x_-NPs/Cs_0.175_FA_0.750_MA_0.075_Pb(I_0.88_Br_0.12_)_3_/PC_61_BM/ZnO NPs/Ag	0.1	23.13	1.06	65.59	16.08	[103]
2022	ITO/NiO_x_-NPs/Cs_0.17_FA_0.83_Pb(I_0.87_Br_0.13_)_3_/PCBM/ZnO NPs/Ag	0.1	22.92	1.06	70.33	17.05	[104]
2022	ITO/NiO_x_/CsFAPbI_3-x_Cl_x_/PCBM/BCP/Cu	0.14	23.72	1.04	70.00	17.33	[101]
2022	ITO/SnO_2_/FA_0.91_Cs_0.09_PbI_3_/Spiro-OMeTAD/Au/Ag	0.2	23.00	1.02	74.66	17.45	[105]
2022	ITO/Poly-TPD/MAPbI_3_/PCBM/BCP/Ag	0.25	21.80	1.08	75.00	17.50	[106]
2022	ITO/SnO_2_/CsMAFA-Perovskite/Spiro-OMeTAD/Ag	0.1	22.09	1.08	76.01	18.13	[107]
2022	FTO/SnO_2_/Cs_0.15_FA_0.85_Pb(I_0.83_Br_0.17_)_3_/Spiro-OMeTAD/Au	0.09	22.56	1.09	77.01	18.94	[108]
2022	FTO/SnO_2_/FAMACs-perovskite/Spiro-OMeTAD/Au	0.09	21.70	1.108	79.80	19.20	[109]
2022	ITO/2PACz/FAMACs-perovskite/LiF/C_60_/SnO_2_/Ag	0.16	19.60	1.20	78.70	19.52	[110]
2022	FTO/c-TiO_2_/MAPbI_3_/PCBM/Au	0.1	24.64	1.13	76.99	21.44	[111]
2022	FTO/NiO_x_/FACs-perovskite/PCBM/BCP/Ag	0.09	24.80	1.16	81.40	23.40	[112]
2023	PET/ITO/SnO_2_/3D–2D perovskite/Carbon	0.64	15.3	0.99	47.00	7.70	[113]
2023	FTO/c-TiO_2_/m-TiO_2_/(Cs_0.05_(MA_0.17_FA_0.83_)_0.95_Pb(I_0.83_Br_0.17_)_3_/CIS/Carbon	\	18.33	0.93	58.00	9.93	[114]
2023	FTO/NiO_x_/MAPbI_3_/PC_61_BM/TBAOH/Ag	0.75	10.10	2.11	63.30	13.54	[115]
2023	ITO/PFTPA/MAPbI_3_/PC_61_BM/PEIE/Ag	0.1	19.50	1.08	65.70	13.84	[116]
2023	FTO/NiO_x_/MAPbI_3_/PC_61_BM/TBAOH/Ag	0.09	20.34	1.00	68.03	13.86	[115]
2023	ITO/c-TiO_2_/M-TiO_2_/(FA_0.85_Cs_0.15_)Pb(I_0.97_Br_0.03_)_3_/Spiro-OMeTAD/Au	1.0	22.40	1.02	62.00	14.40	[117]
2023	ITO/NiO_x_/MeO-2PACz/Cs_0.175_FA_0.750_MA_0.075_Pb(I_0.880_Br_0.120_)_3_/PC_61_BM/ZnO/Cu	8.64	4.70	6.24	60.90	17.66	[118]
2023	ITO/2PACz/FA_0.8_MA_0.2_Pb(I_0.8_Br_0.2_)_3_/LiF/C_60_/BCP/Cu	0.16	21.85	1.088	75.00	17.83	[119]
2023	ITO/2PACz/Cs_0.1_(FA_0.83_MA_0.17_)_0.9_(I_0.83_Br_0.17_)_3_/C_60_/BCP/Au	0.105	21.10	1.09	71.00	18.20	[120]
2023	FTO/SnO_2_/MA_0.30_FA_0.70_Pb(I_0.84_,Br_0.16_)_3_/Spiro-OMeTAD/Au	0.09	22.0	1.14	79.00	19.80	[121]
2023	FTO/2PACz/CsMAFA-perovskite/LiF/C_60_/SnO_2_/Ag	0.16	20.89	1.21	78.80	19.90	[122]
2023	FTO/SnO_2_/Cs_0.15_FA_0.85_Pb(I_0.83_Br_0.17_)_3_/Spiro-OMeTAD/Au	0.09	23.56	1.09	77.00	19.95	[123]
2023	FTO/PTAA/Al_2_​O_3_​/FA_0.83_Cs_0.17_PbI_3_/C_60_​/SnO_2_​/Ag	1.0	22.88	1.11	80.00	20.17	[124]
2023	ITO/SnO_2_/MAPbI_3_/Spiro-OMeTAD/Au	0.049	23.77	1.06	82.60	20.87	[125]
2023	FTO/c-TiO_2_/F-LYS-S-MAPbI_3_/Spiro-OMeTAD/Au	0.1	24.80	1.104	77.00	21.08	[126]
2023	ITO/MeO-2PACz/FAPbI_3_/LiF/C_60_/SnO_2_/Cu	0.096	24.92	1.088	83.10	22.54	[127]
2024	PET/IMI/SnO_2_/FACsPbBr_3_/PEDOT/Carbon	\	8.43	1.47	72.30	8.97	[128]
2024	FTO/SnO_2_/MAPbI_3_/P3HT/Ag	0.1	17.25	0.981	71.00	11.99	[129]
2024	ITO/PTAA/FA_0.78_Cs_0.22_Pb(I_0.85_Br_0.15_)_3_/C_60_/BCP/Al	0.0625	18.80	1.01	62.80	12.11	[130]
2024	PET/ITO/SnO_2_/FAMACs-perovskite/Spiro-OMeTAD/Au	\	21.31	1.09	72.90	16.87	[131]
2024	ITO/NiO_x_/Me-4PACz/Cs_0.175_FA_0.750_MA_0.075_Pb(I_0.880_Br_0.120_)_3_/PCBM/ZnO/Ag	2.7	4.29	6.13	66.09	17.42	[132]
2024	ITO/SnO_2_/MAPbI_3_/Spiro-OMeTAD/Au	0.2	23.48	1.06	75.00	18.57	[133]
2024	FTO/c-TiO_2_/CsPbI_2.77_Br_0.23_/Spiro-OMeTAD/Au	0.1	20.91	1.131	80.57	19.05	[134]
2024	FTO/c-TiO_2_/CsPbI_2.77_Br_0.23_/Spiro-OMeTAD/Au	0.453	24.32	1.05	79.28	20.27	[135]
2024	FTO/SnO_2_/FA_0.92_MA_0.08_PbI_2.76_Br_0.24_/ammonium salt/Spiro-OMeTAD/Au	0.16	24.90	1.17	82.70	24.10	[136]
2025	ITO/2PACz/CsFAMA-perovskite/C_60_/BCP/Au	0.0105	21.90	1.13	77.00	19.10	[137]
2025	ITO/NiO_x_/MeO-2PACz/Cs_0.175_FA_0.750_MA_0.075_(I_0.88_Br_0.12_)_3_/PC_61_BM/ZnO/Cu	2.7	6.90	4.30	68.12	20.26	[138]
2025	ITO/NiO_x_/MeO-2PACz/Cs_0.175_FA_0.750_MA_0.075_(I_0.88_Br_0.12_)_3_/PC_61_BM/ZnO/Cu	0.1	25.38	1.15	72.56	21.32	[138]
2025	FTO/NiO_x_/Me-4PACz/Cs_0.05_(MA_0.05_FA_0.95_)_0.95_Pb(I_0.95_Br_0.05_)_3_/C_60_/BCP/Cu	1.0	24.72	1.13	82.82	23.13	[139]
2025	ITO/NiO_x_/2PACz/Cs_0.05_FA_0.95_PbI_3_/PEAI/C_60_/BCP/Cu	0.062	25.75	1.11	84.33	24.20	[25]
2025	ITO/PTAA/FA_0.83_Cs_0.17_PbI_3_/C_60_/BCP/Cu	0.07	25.02	1.16	84.69	24.58	[140]

**Table 2:** Module configurations and *J–V* scan photovoltaic parameters of the slot-die-coated PSMs

Year	Module configuration	Active area (cm^2^)	*Jsc* (mA cm^2^) /*I* (mA)	*Voc* (V)	*FF* (%)	*Eff* (%)	Refs
2015	ITO/ZnO/MAPbI_3_/doped P3HT/Ag	47.30	10.2	4.35	51.10	4.56	[59]
2017	ITO/TiO_2_/MAPbI_3-x_Cl_x_/Spiro-OMeTAD/Au	168.75 (Ap)	17.30	21.2	67.90	10.00	[73]
2017	FTO/ZnO/MAPbI_3_/Carbon	17.60	3.25	6.14	53.00	10.60	[45]
2018	ITO/PEDOT:PSS/MAPbI_3_/C_60_/PCBM/BCP/Ag	10	4.10	3.8	52.00	8.30	[78]
2019	FTO/NiO_x_/FA_0.85_MA_0.15_Pb(I_0.85_Br_0.15_)_3_/G-PCBM/BCP/Ag	35.80	65.7	10.80	71.50	14.17	[141]
2020	FTO/TiO_2_/SnO_2_/FA_x_Cs_1-x_PbI_x_Cl_3-x_/BJ-GO/TFB/Cr/Au	35.80	2.04	10.99	72.31	16.21	[142]
2020	FTO/TiO_2_/FA_0.91_Cs_0.09_PbI_3_/PC_61_BM)/Ag	10.20	45.6	5.44	76.40	18.60	[91]
2021	FTO/SnO_2_/Cs_0.16_FA_0.84_Pb(I_0.88_Br_0.12_)_3_/Spiro-OMeTAD/Au	52	9.2	12.03	54.90	11.60	[40]
2021	FTO/c-TiO_2_/m-TiO_2_/Cs_0.17_FA_0.83_Pb(I_0.83_Br_0.17_)_3_/Spiro-OMeTAD/Au	12 (Ap)	1.42	6.60	70.20	13.09	[95]
2021	FTO/c-TiO_2_-SnO_2_/Cs_0.05_MA_0.4_FA_0.55_Pb(I_0.96_Br_0.04_)_3_/Spiro-OMeTAD/Au	12 (Ap)	3.16	6.48	74.10	15.20	[97]
2021	FTO/c-TiO_2_/m-TiO_2_/GO-K/Cs_0.1_FA_0.9_Pb(I_0.94_B_r0.06_)_3_/Spiro-OMeTAD/Au	16	44.31	8.22	88.00	16.10	[96]
2021	FTO/NiMgLiO/FA_0.83_Cs_0.17_PbI_2.83_Br_0.17_/LiF/C_60_/BCP/Bi/Ag	20.77	428.5	1.0845	74.30	16.63	[43]
2021	FTO/SnO_2_/3D perovskite (FA_0.83_Cs_0.17_PbI_3-x_Cl_x_)/2D perovskite/Spiro-OMeTAD/Au	65	1.67	15.35	76.10	19.54	[143]
2022	FTO/SnO_2_/Cs_0.15_FA_0.85_Pb(I_0.83_Br_0.17_)_3_/Spiro-OMeTAD/Au	57.50	100	14.23	64.86	16.22	[108]
2022	FTO/SnO_2_/FAMACs-perovskite/Spiro-OMeTAD/Au	37.60	1.31	17.61	73.50	18.10	[109]
2022	FTO/NiO_x_/FACs-perovskite/PCBM/BCP/Ag	174	198.9	20.74	78.40	18.60	[112]
2023	ITO/NiO_x_/FA_0.8_Cs_0.2_Pb(I_0.94_Br_0.06_)_3_/LiF/C_60_/BCP/Cu	784 (Ap)	260	53.66	65.00	11.65	[144]
2023	ITO/MeO-2PACz/FAPbI_3_/LiF/C_60_/SnO_2_/Cu	12.70	3.06	8.36	66.80	17.10	[127]
2023	ITO/2PACz/Cs_0.1_(FA_0.83_MA_0.17_)0.9(I_0.83_Br_0.17_)_3_/C_60_/BCP/Au	12.96	2.1	9.51	68.00	17.20	[120]
2023	FTO/PTAA/Al_2_​O_3_​/FA_0.83_Cs_0.17_PbI_3_/C_60_​/SnO_2_​/Ag	60.84 (Ap)	105.37	14.08	72.00	17.56	[124]
2023	FTO/SnO_2_/Cs_0.15_FA_0.85_Pb(I_0.83_Br_0.17_)_3_/FABP/Spiro-OMeTAD/Au	58.50	109	13.70	75.04	19.28	[123]
2023	ITO/SnO_2_/(FAPbI_3_)_0.95_(MAPbBr_3_)_0.05_/PEAI/Spiro-OMeTAD/Au	10 (Ap)	3.98	6.69	78.00	20.98	[145]
2024	PET/IMI/SnO_2_/FACsPbBr_3_/PEDOT/Carbon	16.84	15.11	9.21	63.70	5.30	[128]
2024	FTO/SnO_2_/MAPbI_3_/P3HT/Ag	64.80	134.8	7.05	53.00	7.77	[129]
2024	PET/ITO/SnO_2_/FAMACs-perovskite/Spiro-OMeTAD/Au	94.60	1.05	18.82	56.99	11.25	[131]
2024	ITO/PTAA/FA_0.25_MA_0.75_PbI_3_/C_60_/BCP/Ag	14.40	105.3	3.15	63.87	14.70	[135]
2024	FTO/TiO_2_/SnO_2_/Cs_0.17_FA_0.83_PbI_3_/Spiro-OMeTAD/Au	80 (Ap)	1.55	14.00	74.10	16.10	[146]
2024	FTO/SnO_2_/FACs-perovskite/Spiro-OMeTAD/Au	802 (Ap)	0.54	44.37	73.40	17.59	[147]
2024	FTO/NiO_x_/FA_0.91_Cs_0.09_PbI_3_/PMI-FPMI/C_60_/TPBi/Cr/Cu	171.50	132.56	30.67	79.01	18.73	[148]
2024	FTO/NiO_x_/Cs_0.05_MA_0.16_FA_0.79_Pb(Br_0.16_I_0.84_)_3_/L-AA/C_60_/BCP/Cu	57.30	2.18	11.80	74.50	19.17	[149]
2024	ITO/DCMA-SnO_2_/CsFAMA-perovskite/Spiro-OMeTAD/Au	23.25	3.026	9.189	74.12	20.61	[150]
2024	FTO/TiO_2_/SnO_2_/Cs_0.05_MA_0.05_FA_0.9_PbI_3_/Spiro-OMeTAD/Au	1160 (Ap)	332	92.49	77.90	20.62	[44]
2024	FTO/MeO-2PACz/FAMACs-perovskite/PCBM/BCP/Ag	1160	2.91	92.391	78.40	21.10	[151]
2024	FTO/SnO_2_/FA_0.92_MA_0.08_PbI_2.76_Br_0.24_/ammonium salt/Spiro-OMeTAD/Au	10 (Ap)	40.20	6.94	78.30	21.90	[136]
2024	FTO/NiO_x_/Me-4PACz/FA_0.93_MA_0.02_Cs_0.05_PbI_2.94_Br_0.06_/PEACl/PCBM/SnO_2_/Cu	14.61 (Ap)	58.8	7.098	79.50	22.73	[152]
2024	FTO/TiO_2_/SnO_2_/2D–3D FAPbI_3_/Spiro-OMeTAD/MoOx/Cu	715.10 (Ap)	398.24	49.51	82.68	22.80	[27]
2025	FTO/NiO_x_/PTAA/FA_0.9_Cs_0.1_PbI_3_/C_60_/SnO_2_/ITO/Cu	7906 (Ap)	0.75	206.05	76.55	14.96	[153]
2025	ITO/PTAA/FA_0.83_Cs_0.17_PbI_3_/C_60_/BCP/Cu	260 (Ap)	0.9	28.40	66.56	17.02	[140]
2025	ITO/2PACz/CsFAMA-perovskite/C_60_/BCP/Au	12.96 (Ap)	31.40	9.98	74.00	17.90	[137]
2025	ITO/NiO_x_/Me-4PACz/Al_2_O_3_/Cs_0.05_FA_0.95_PbI_3_/PCBM/BCP/Ag	642 (Ap)	401.08	38.15	77.53	18.48	[154]
2025	ITO/NiO_x_/2PACz-MeO-4PACz/FAPbI_3_-0D(Tpy)_2_PbI_6_/PCBM/C_60_/SnO_2_/CrAu	785 (Ap)	0.508	50.10	77.00	19.60	[155]
2025	FTO/NiO_x_/Me-4PACz/@BCF/FA_0.95_Cs_0.05_PbI_3_/PDI/C_60_/BCP/Cu	20,000 (Ap)	2.812	182.00	78.35	20.05	[156]
2025	FTO/NiO_x_/Me-4PACz/Cs_0.05_(MA_0.05_FA_0.95_)_0.95_Pb(I_0.95_Br_0.05_)_3_/C_60_/BCP/Cu	56.50 (Ap)	2.19	12.21	80.77	21.50	[138]
2025	ITO/NiO_x_/2PACz/Cs_0.05_FA_0.95_PbI_3_/PEAI/C_60_/BCP/Cu	15.64	3.17	8.90	77.28	21.84	[25]
2025	FTO/TiO_2_/SnO_2_/2D–3D FAPbI_3_/Spiro-OMeTAD/Au	663 (Ap)	303.01	59.01	81.96	22.10	[157]
2025	ITO/SnO_2_-CIT/Cs_0.03_FA_0.97_PbI_3_/Spiro-OMeTAD/Au	23.26	3.27	9.50	73.04	22.70	[26]
2025	ITO/MeO-2PACz/Al_2_O_3_/Cs_0.01_(FA_0.97_MA_0.03_)_0.99_Pb(I_0.97_Br_0.03_)_3_/PEAI/LiF/C_60_/BCP/Ag	100 (Ap)	2.49	11.90	81.20	24.00	[158]

It is widely recognized that the quality of the perovskite active layer is the most critical factor in determining the performance of PSCs [62, 63]. Thus, attaining high-quality perovskite films is essential for the performance enhancement of PSCs/PSMs. The following sections discuss four key engineering strategies developed to enhance slot-die-coated perovskite films: process engineering, solvent engineering, additive engineering, and interface engineering. These approaches primarily aim to suppress or prevent the uneven distribution of defect sites in perovskite films, thereby enhancing device efficiency and stability.

### Stability Assessment: Status, Challenges, and a Call for Standardization

While Tables 1 and 2 summarize remarkable progress in the efficiency of slot-die-coated PSCs and PSMs, the reported long-term stability data exhibit significant disparities, severely hindering objective comparison and reliability assessment of different technological pathways. Currently, the stability testing conditions (e.g., light intensity, spectrum, temperature, humidity, bias, encapsulation status) adopted in most cited high-efficiency module papers lack uniformity [159, 160]. For instance, some studies test only under room temperature, inert atmosphere, or dark storage, while others employ more stringent but varied “light + heat” or “damp heat” aging tests. This “à la carte” approach to testing, while demonstrating device potential under specific conditions, leads to several critical issues: (1) Lack of Comparability: Due to differing protocols, the stability of Device A after 1000 h at 85 °C in the dark cannot be meaningfully compared to that of Device B after 500 h at 85 °C/85% RH, or Device C after 600 h of continuous maximum power point tracking (MPPT) illumination. (2) Inadequate Representation of Real-World Conditions: Many tests fail to simulate the synergistic multistress factors (coupled light, heat, moisture, electrical bias, oxygen) encountered in actual outdoor operation, potentially deviating from predicting real-world device lifetime. (3) Limited Industrial Reference Value: The proliferation of disparate standards in testing standards makes it difficult for investors and manufacturers to assess the true risk and durability of the technology, increasing uncertainty for industrialization.

The international community has long recognized this issue and established the International Summit on Organic Photovoltaic Stability (ISOS) series of protocols (e.g., ISOS-L, ISOS-D, ISOS-O) [161, 162]. These protocols provide a detailed, unified framework for aging tests of optoelectronic devices under different stress conditions. We strongly advocate that future research on slot-die-coated perovskite modules, especially those reporting high efficiencies, must explicitly adhere to or at least benchmark against relevant ISOS protocols for stability testing and reporting. This is not only a requirement for research rigor but also a cornerstone for fostering the healthy and credible development of the entire field.

To provide concrete guidance, we propose a tiered approach to stability reporting based on the research stage: (1) Fundamental Material/Process Study: At minimum, report stability under ISOS-D-1 (dark storage, controlled environment) and ISOS-L-1 (continuous illumination, room temperature). (2) High-Efficiency Device/Module Demonstration: Must include ISOS-L-2 (continuous illumination with maximum power point tracking at elevated temperature, e.g., 65 or 85 °C) and ISOS-D-2 (damp heat, e.g., 85 °C/85% RH) data. Encapsulation status must be clearly stated. 3. Pre-commercial Module Validation: Should incorporate ISOS-O-1 (outdoor testing) alongside rigorous ISOS-L-2 and ISOS-D-2 testing to validate real-world performance and reliability.

Adopting such a standardized, tiered reporting framework will enable meaningful cross-comparison, build investor confidence, and accelerate the identification of truly robust technological pathways. Furthermore, to more effectively guide stability improvement, it is necessary to systematically delineate the typical failure modes of slot-die-coated devices from the perspective of their unique processing characteristics. Compared to spin-coated films, slot-die-coated films exhibit distinct features in defect distribution (e.g., stripe-like inhomogeneity along the coating direction), residual stress state, and large-area interfacial uniformity, which may lead to differentiated degradation behaviors. Major failure modes can be categorized as [163–166]: (1) Moisture-induced degradation: Hydration reactions preferentially initiate at pinholes or weak grain boundaries, with crack propagation potentially accelerated by film stress; (2) Ion migration and phase segregation: Under the influence of large-area electric fields, ion migration along defect channels and subsequent halide phase separation may be more pronounced; (3) Interfacial delamination: Local detachment at the large-area interface between the perovskite and charge transport layers may occur due to differential drying kinetics or interfacial energy mismatch, particularly under thermal cycling stress; (4) Metal electrode corrosion: Moisture and oxygen ingress from edges or laser-scribed P2/P3 channels, synergistically with migrating halide ions, accelerate the electrochemical corrosion of electrodes (especially Ag or Cu). Future stability research should combine in situ imaging techniques (e.g., PL, thermal imaging) with micro-area analysis of failure sites (e.g., ToF–SIMS, micro-XPS) to clarify the intrinsic relationship between these failure modes and slot-die processing parameters, thereby enabling a leap from “generic” stabilization strategies to “process-tailored” ones.

## Key Engineering Strategies for High-Quality Slot-Die-Coated Perovskite Films

### Process Engineering

#### One-Step Process

In the one-step process, the perovskite ink, containing lead precursors and organic salts with a specific stoichiometric ratio is directly cast onto the substrate. Unlike spin coating, which enables rapid solvent evaporation via centrifugal force, slot-die coating involves slower drying kinetics. This reduced evaporation rate not only significantly suppresses crystal growth but also lowers nucleation density of the resulting films. To address this, techniques including in situ annealing [39, 40], gas blowing [40, 41], anti-solvent extraction [42, 43], vacuum flashing [44, 45], and 3D laminar flow-assisted crystallization [152] have been employed to accelerate the solvent removal. Paradoxically, excessively rapid solvent volatilization frequently induces Marangoni-driven dewetting, resulting in non-uniform coverage with pinholes and thickness variations in the final dried films [167, 168]. Thus, precise control over perovskite nucleation and crystallization during drying is the fundamental requirement for high-quality slot-die-coated films.

**Fig. 5:**
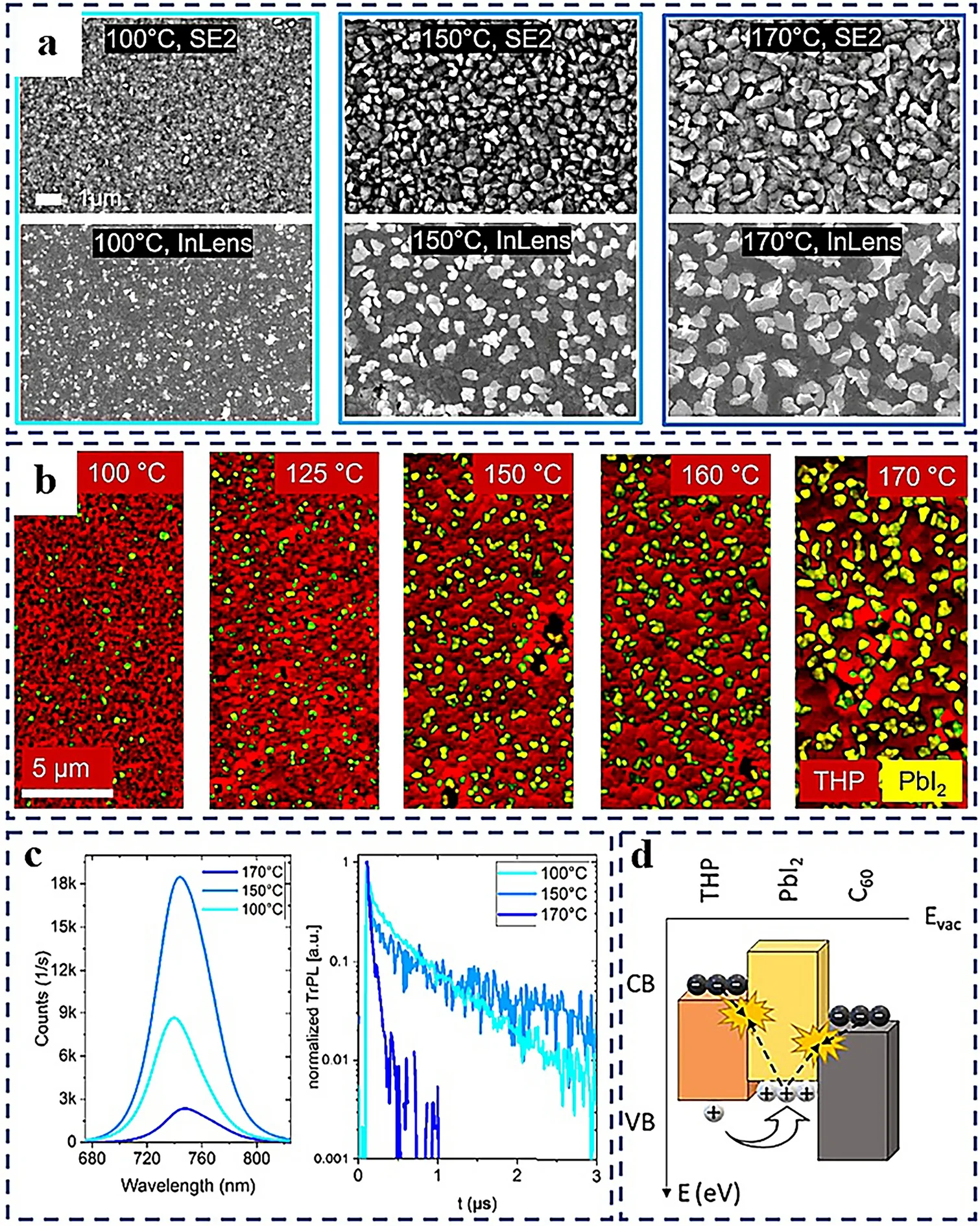
**a** Top view SEM images of perovskite films showing morphology (top row, SE2 detector) and surface composition distribution (bottom row, InLens detector). Scale bar applies to all panels. Reproduced with permission from Ref. [110]. Copyright 2022, American Chemical Society. **b** Cathodoluminescence (CL) composite maps showing triple-halide perovskite (THP) luminescence (750 ± 50 nm, red) and PbI_2_ emission (500 ± 50 nm, yellow). Reproduced with permission from Ref. [122]. Copyright 2023, American Chemical Society. **c** PL spectra and time-resolved PL transients. Reproduced with permission from Ref. [110]. Copyright 2022, American Chemical Society. **d** Schematic of interfacial PbI_2_ hindering charge transport and increasing recombination. Reproduced with permission from Ref. [122]. Copyright 2023, American Chemical Society

Start with the thermal annealing, despite the grain size increased with higher annealing temperatures, pronounced PbI_2_ appeared in 170 °C annealed films (Fig. 5a), leading to severe non-radiative recombination (Fig. 5c) [110]. Follow-up study corroborated this via cathodoluminescence imaging, revealing platelet-like PbI_2_ precipitates at elevated temperatures (Fig. 5b). These insulating phases at grain boundaries increased series resistance by impeding charge transport and intensified interfacial recombination (Fig. 5d), thereby reducing open-circuit voltage (*Voc*) and fill factor (*FF*) [122]. Beyond annealing temperature, annealing protocol critically governed the surface topography of films: gradient annealing halved surface roughness compared to single-temperature protocols [69]. Additionally, prolonged annealing (> 50 min) ensured complete solvent evaporation and precursor conversion, suppressing residual intermediate phases [77].

**Fig. 6:**
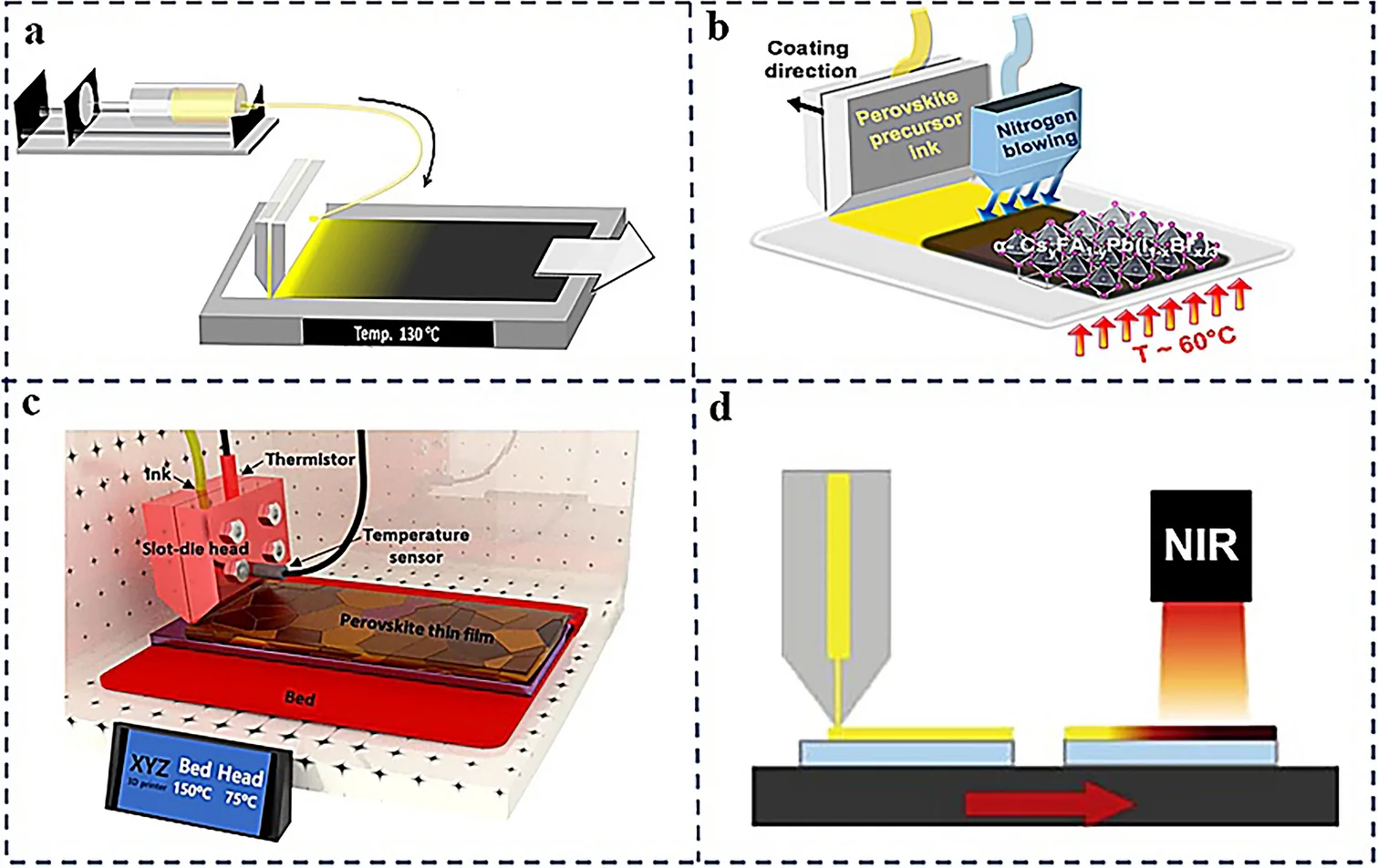
**a** Integrated schematic of slot-die coating with in situ annealing. Reproduced with permission from Ref. [39]. Copyright 2020, American Chemical Society. **b** Schematic of the slot-die coating process integrated with gas quenching and substrate heating. Reproduced with permission from Ref. [40]. Copyright 2020, Elsevier. **c** Schematic of the slot-die coating system with an integrated hot coating head. Reproduced with permission from Ref. [104]. Copyright 2022, Elsevier. **d** Schematic diagram of slot-die-coated perovskite layer fabricated by NIR heating. Reproduced with permission from Ref. [88]. Copyright 2020, John Wiley and Sons

Preheated substrates have been extensively employed in slot-die coating to enable in situ solvent removal. Notably, devices fabricated on substrates annealed at 130 °C matched the PCEs of spin-coated counterparts (Fig. 6a) [39]. Kamaraki et al*.* further optimized the substrate temperature for MAPbI_3_ perovskite films, identifying 55 °C as optimal for device performance [74]. Although substrate heating promoted smooth films with enhanced crystallinity, it concurrently induced dewetting phenomena, which compromised film coverage. To address this, gas quenching with nitrogen (flow rate: 100 L min^–1^) was integrated into the heated slot-die system (Fig. 6b). This approach yielded dense, pinhole-free films, enabling FTO/SnO_2_/Cs_0.16_FA_0.84_Pb(I_0.88_Br_0.12_)_3_/Spiro-OMeTAD/Au devices (active area: 52 cm^2^) to reach a PCE of 11.60% [40]. Interestingly, film coverage progressively improved with increasing substrate temperature under nitrogen flow. However, a distinct morphological transition occurred above 70 °C, characterized by the transformation of needlelike grains into rounded crystals, potentially attributable to altered crystallization kinetics [41]. To minimize the influence of substrate temperature on crystallization kinetics, Rezaee et al*.* employed a room-temperature substrate coupled with N_2_ gas purging. This approach effectively induced a transition in the film formation mechanism from crystal growth-dominated (CGD) to nucleation-dominated (ND), thereby achieving thin-film quality comparable to spin-coated counterparts in high-performance optoelectronic devices [106]. Alternatively, You et al*.* established a low-temperature substrate growth (LTSG) methodology to synthesize large-area homogeneous perovskite films by modulating crystallization kinetics. This strategy effectively mitigated the impact of ambient temperature fluctuations on film quality, resulting in only an ~ 0.75% reduction in absolute PCE for each order-of-magnitude increase in device area [152].

To further enhance perovskite film quality, Cotella et al. integrated a heated head and gas knife into the slot-die coating process [67]. The heated head suppressed premature PbI_2_ crystallization, while the thermal gradient established by substrate heating and cold gas quenching induced horizontal crystal growth, thereby reducing film roughness. Extending this strategy, Seo et al*.* systematically modulated coating head and substrate temperatures to regulate perovskite crystallization (Fig. 6c). They demonstrated that preheating the coating head (75 °C) inhibited nucleation and promoted grain growth, producing large-grained films. Under optimal conditions (coating head: 75 °C, substrate: 150 °C), PSMs reached PCEs approaching 17% [104]. Beyond conventional heating, near-infrared irradiation (NIR) has gained attention for its high thermal efficiency (Fig. 6d). NIR heating significantly accelerated solvent evaporation, enabling ultrafast processing (< 20 s) [88]. Recently, Chen et al. reported a high-power blue laser-based rapid annealing technique, which enables high-quality crystallization of large-area thin films within approximately 20 s. Fabricated via this process, both rigid modules (100 cm^2^ in area) and their flexible counterparts achieved certified power conversion efficiencies of 24.00% and 20.70%, respectively [158].

**Fig. 7:**
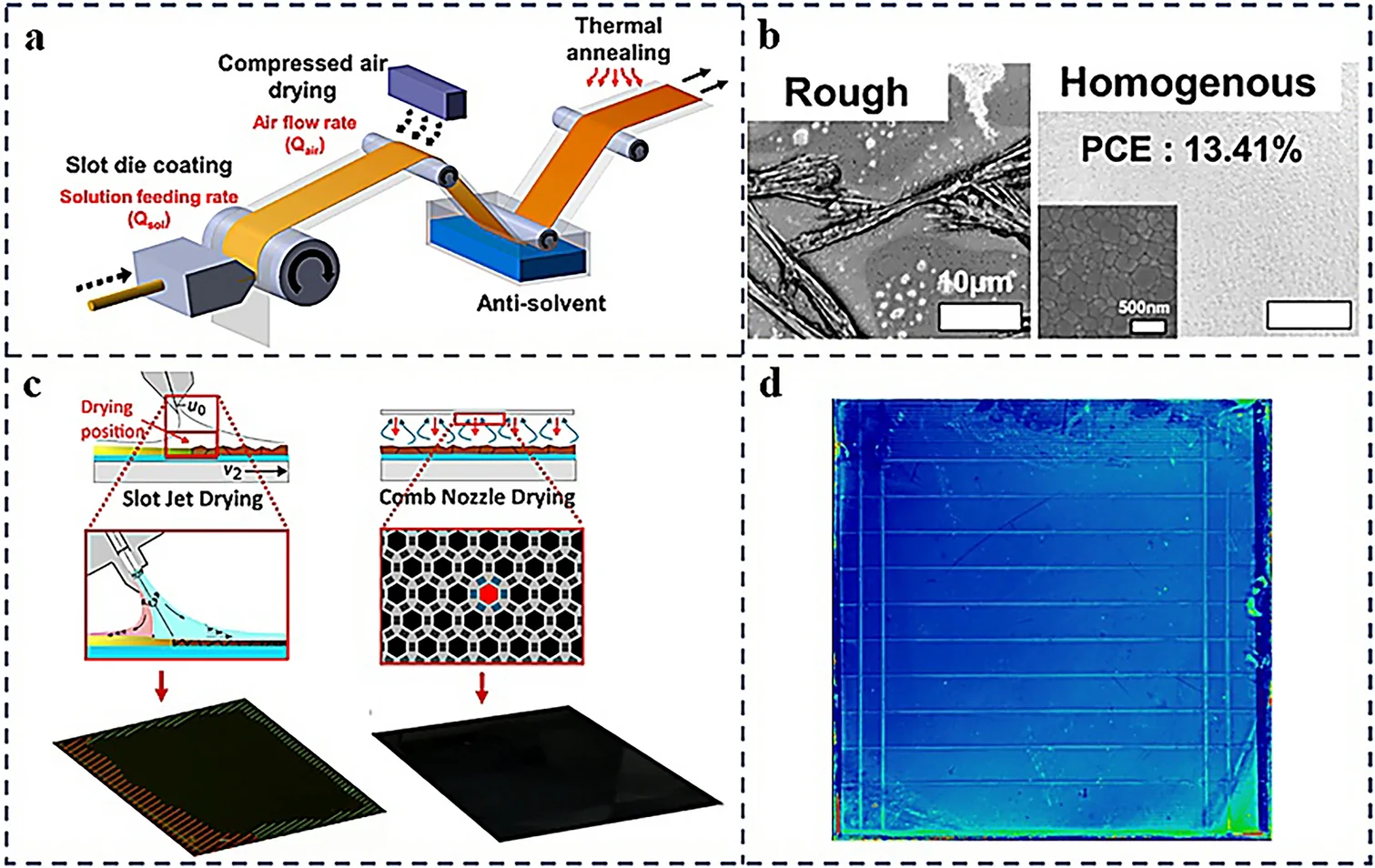
**a** Schematic of the R2R process of the perovskite layer. **b** Photographic and SEM images of perovskite films. Reproduced with permission from Ref. [42]. Copyright 2021, American Chemical Society. **c** Schematic of the novel, alternative drying system, comb nozzle (CN) drying method and **d** PL measurement of one exemplary module. Reproduced with permission from Ref. [137]. Copyright 2025, John Wiley and Sons

Additionally, in one-step slot-die coating, the solution feeding rate (Q_sol_) and gas quenching rate (Q_air_) predominantly dictate perovskite nucleation kinetics and crystallization quality by modulating precursor supersaturation and solvent evaporation dynamics. Precise compatibility between Q_sol_ and Q_air_ is critical—incompatible ratios induce pinholes and inhomogeneous crystallization. Ham et al. systematically optimized these parameters in a R2R process with toluene anti-solvent quenching, fabricating pinhole-free triple-cation perovskite films achieving 13.41% PCE (Fig. 7a, b) [42]. However, conventional linear nozzles constrain drying uniformity control [120]. To overcome this limitation, Geistert et al*.* developed a two-dimensional comb nozzle (CN) to replace conventional linear nozzles (Fig. 7c). This CN eliminated spatial solvent evaporation gradients through bidirectional gas flow, significantly improving the drying uniformity of perovskite wet films. Consequently, CN-processed perovskite modules exhibited enhanced photoluminescence uniformity (Fig. 7d) and 90% fabrication yield with PCE > 19% [137]. Interestingly, a dual-solution (MAPbI_3_/FAPbI_3_) feeding system has been developed, thus allowing the preparation of tunable color perovskite films [119].

On the core challenges of one-step deposition, the fundamental contradiction lies in a formidable trilemma of “kinetics–morphology–uniformity.” Foremost is the kinetic competition between solvent evaporation and crystallization nucleation/growth. Rapid evaporation promotes high-density nucleation but risks inducing rheological instabilities and film defects, whereas slow evaporation leads to coarse grains and porous structures. The absence of a universal kinetic theoretical model forces process optimization to rely on trial-and-error, local parameter tuning, resulting in a notoriously narrow processing window and making uniformity control in large-area fabrication a daunting challenge. Secondly, the role and origin of residual PbI_2_ remain a contentious scientific enigma. While its presence is common, the cause of its segregation—whether due to stoichiometric deviation, solvation disparity, or interfacial energy driving—is still debated. Its impact is dualistic, ranging from beneficial defect passivation to detrimental recombination centers, and critically, clear “safe thresholds” or “functional windows” for quantitative guidance are lacking. A deeper understanding requires atomistic-scale in situ studies to map the transformation pathway from PbI_2_–solvent complexes to the perovskite phase under coating-relevant drying kinetics. Ultimately, scale-up exacerbates these contradictions. Inconsistent drying fronts and edge effects translate directly into spatial heterogeneity in film morphology and phase composition, constituting an engineering science hurdle that is difficult to overcome by hardware modifications alone.

#### Two-Step Process

Compared with the one-step process, the two-step method enables excellent morphological control and high reproducibility [169]. A typical two-step deposition protocol comprises the following sequential steps: (1) PbI_2_ deposition, defining final thickness and coverage, and (2) conversion to perovskite via reaction with organic/inorganic halide solutions [170]. However, incomplete conversion typically leaves trace residual PbI_2_ at buried interfaces. This Pb-rich interfacial phase significantly compromises device performance by impeding charge transport and attenuating light absorption, as widely documented [171–173]. Therefore, eliminating unconverted phases is critical for advancing two-step perovskite photovoltaics.

**Fig. 8:**
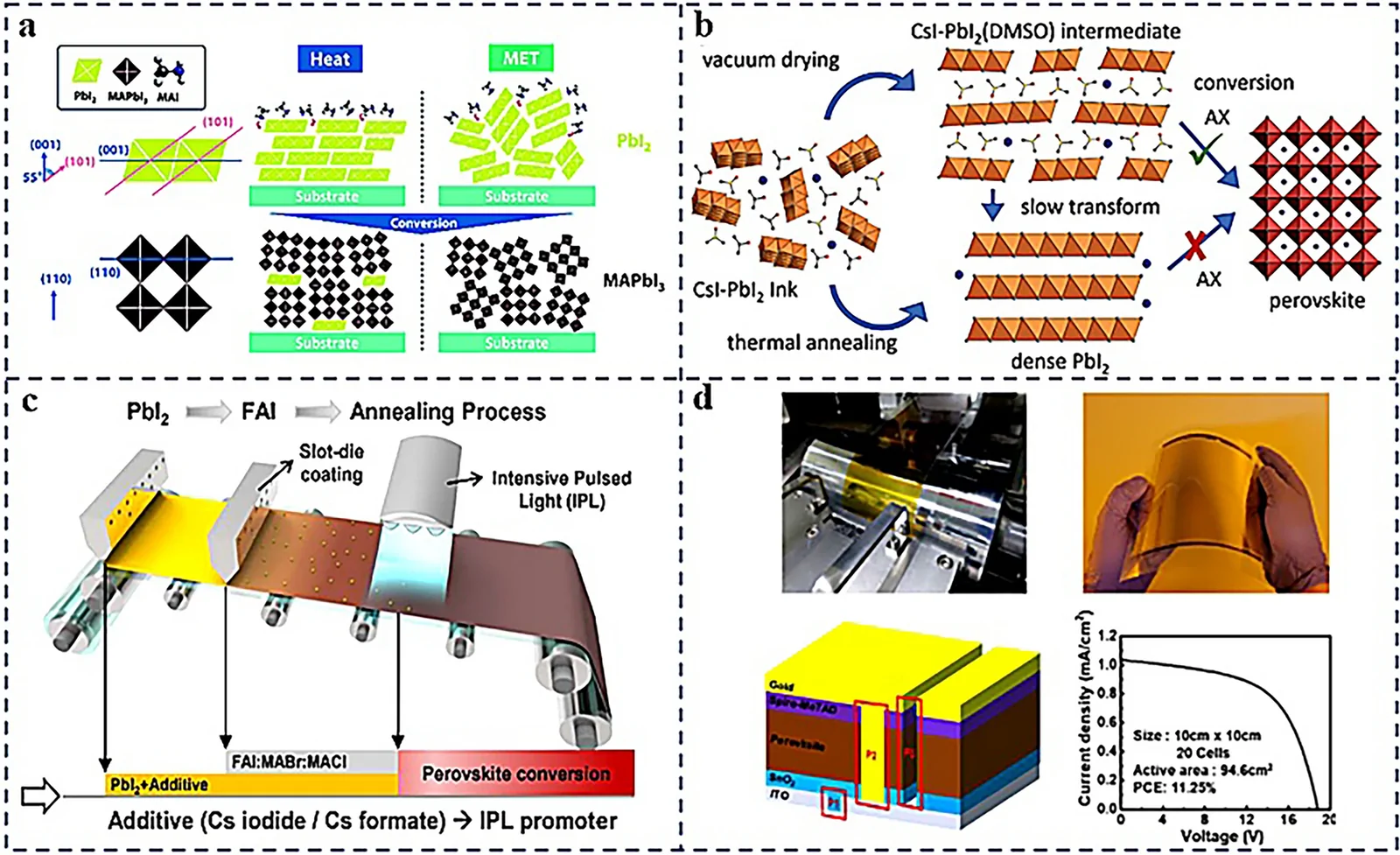
**a** Schematic diagram illustrating the difference in relative orientation between PbI_2_ films (fabricated by MET or heat treatment) and the subsequently formed MAPbI_3_ films. Reproduced with permission from Ref. [82]. Copyright 2018, The Royal Society Chemistry. **b** Sequential perovskite deposition schematic: Vacuum drying yields a porous precursor layer that facilitates subsequent conversion to perovskite, whereas thermal annealing produces a highly dense crystalline PbI_2_ layer, which impedes complete perovskite conversion. Reproduced with permission from Ref. [97]. Copyright 2018, John Wiley and Sons. **c** and **d** Schematic of the R2R two-step process with an IPL and the corresponding flexible module. Reproduced with permission from Ref. [131]. Copyright 2024, The Royal Society Chemistry

PbI_2_ film morphology critically dictates the quality of resultant perovskite films. For instance, maintaining N_2_-blown films in a sealed environment induced cloudy PbI_2_ layers with enlarged pinholes, thereby facilitating methylammonium iodide (MAI) molecular penetration [66]. Alternatively, dimethyl sulfoxide (DMSO) vapor treatment—converting sheetlike PbI_2_ into rodlike architectures to accelerate perovskite conversion kinetics—demonstrated that targeted manipulation of the PbI_2_-DMSO intermediate phase profoundly dictated final perovskite film quality [121]. Similarly, mediator extraction technology (MET), involving DMSO removal from the PbI_2_-DMSO intermediate, generated randomly oriented, highly accessible porous PbI_2_ films (Fig. 8a). This morphology enhanced perovskite conversion efficiency, enabling corresponding devices fabricated by this method to achieve a PCE of 18.80% [82].

Building on morphology control, optimizing PbI_2_ precursor composition is equally pivotal. For CsI-modified precursors, the synergistic combination of gas quenching and substrate annealing generated porous PbI_2_–CsI frameworks. This precisely engineered porosity significantly enhanced conversion kinetics and reaction completeness [107]. Vacuum flashing efficiently extracted DMSO from the PbI_2_–CsI (DMSO) intermediate phase (Fig. 8b), enhancing porosity to facilitate rapid organic cation penetration and enable uniform conversion to pinhole-free perovskites [97]. Concurrently, introducing MA/FA-type additives suppressed ordered PbI_2_ crystallization while promoting perovskite seed formation, thereby resulting in large-grain perovskite films [84]. Notably, Guo et al. demonstrated that trace FAI/MAI/CsI additives induced negligible changes in final morphology yet markedly attenuated PbI_2_ XRD diffraction intensities—providing direct evidence of their critical role in conversion dynamics [90]. This crystallization suppression mechanism was also independently verified by Heo et al. [72].

The second-step coating and annealing processes critically govern PbI_2_–perovskite conversion efficiency. For ITO/ZnO/MAPbI_3_/P3HT/Ag devices, a maximum PCE of 11.96% was obtained through MAI coating at 70 °C [66]. Precise control of the coating gap is essential: excessively thin MAI films (narrow gap) resulted in incomplete PbI_2_ conversion, while overly thick films (wide gap) induced MAI cluster aggregation, which degraded crystallinity and impaired device performance [174]. To circumvent this limitation, a multistep coating protocol employing diluted MAI precursors was developed, achieving near-complete PbI_2_ conversion and yielding 64.80 cm^2^ PSMs with 7.77% PCE [129]. Notably, the use of MAI precursors in ethanol and isopropanol mixed solvents enhanced conversion efficiency owing to reduced ink viscosity and facilitated ink penetration [75]. Further optimization by Vak et al*.* involved immersing unreacted PbI_2_ films in MAI solution for 3 min, followed by N_2_-blown drying; this approach produced films with superior uniformity and lower roughness compared to naturally dried counterparts [59]. Advanced annealing techniques, including NIR and ionized plasma laser (IPL) treatments (Fig. 8c, d) accelerated fabrication while suppressing PbI_2_ residuals [76, 131].

Regarding the unresolved controversies in two-step deposition, the focus revolves around the interconnected chain of “template–conversion–residue.” The primary bottleneck lies in the nano-structural design of the PbI_2_ precursor template, which is trapped in a “porosity versus compactness” dilemma. High porosity is necessary for complete infiltration and conversion by the organic salt solution but may compromise the template’s mechanical integrity and the final perovskite film density. The lack of consensus on the ideal nanostructure reflects an insufficient understanding of its “structure–function” relationship. Furthermore, the microscopic kinetic mechanism of the solid–liquid interfacial conversion reaction remains obscure. Governed by both diffusion and interfacial reaction, the process suffers from a superficial understanding of key intermediate phases (e.g., “perovskite-like” intermediates), their evolution, and manipulation. This makes achieving uniform and complete conversion across large areas a persistent and serious challenge. Particularly critical is that residual PbI_2_ once again emerges as a “double-edged sword.” Trace amounts of well-distributed, crystalline PbI_2_ may provide benign passivation. However, if it exists in “hidden” forms—such as amorphous clusters, nanoscale aggregates, or accumulation at buried interfaces—it often acts as non-radiative recombination centers. The detection blind spots of conventional characterization techniques for such residues can easily lead to misattribution of device performance losses, thereby hindering targeted optimization. Future breakthroughs likely depend on the in situ, multiscale synergistic analysis of the entire chain: “template design—conversion kinetics—qualitative and quantitative analysis of residues.” Advanced techniques like in situ GIWAXS/GISAXS coupled with multimodal spectroscopy are needed to deconvolute the solid-state reaction kinetics and phase evolution in real time.

### Solvent Engineering

As defined in Eq. (1), perovskite ink viscosity and surface tension critically govern the uniformity of wet film during slot-die coating. Fundamentally, final film morphology is dictated by solvent removal dynamics: high-volatility solvents (e.g., diethyl ether, acetonitrile) accelerate evaporation, generating discontinuous films with coarse grains, whereas low-volatility solvents (e.g., DMSO) promote dewetting via prolonged fluidity [175, 176]. Equally pivotal, solvents with strong coordination capability (e.g., dimethylformamide, DMSO) facilitate intermediate phase formation, enabling precise perovskite growth control [177]. This principle is leveraged in mainstream solvents—including dimethylformamide (DMF) [178, 179], DMSO [180, 181], and N-methyl-pyrrolidone (NMP) [182, 183]—where binary or ternary blends synergistically enhance crystallization kinetics and uniformity.

#### High-Boiling-Point Solvent Strategy

**Fig. 9:**
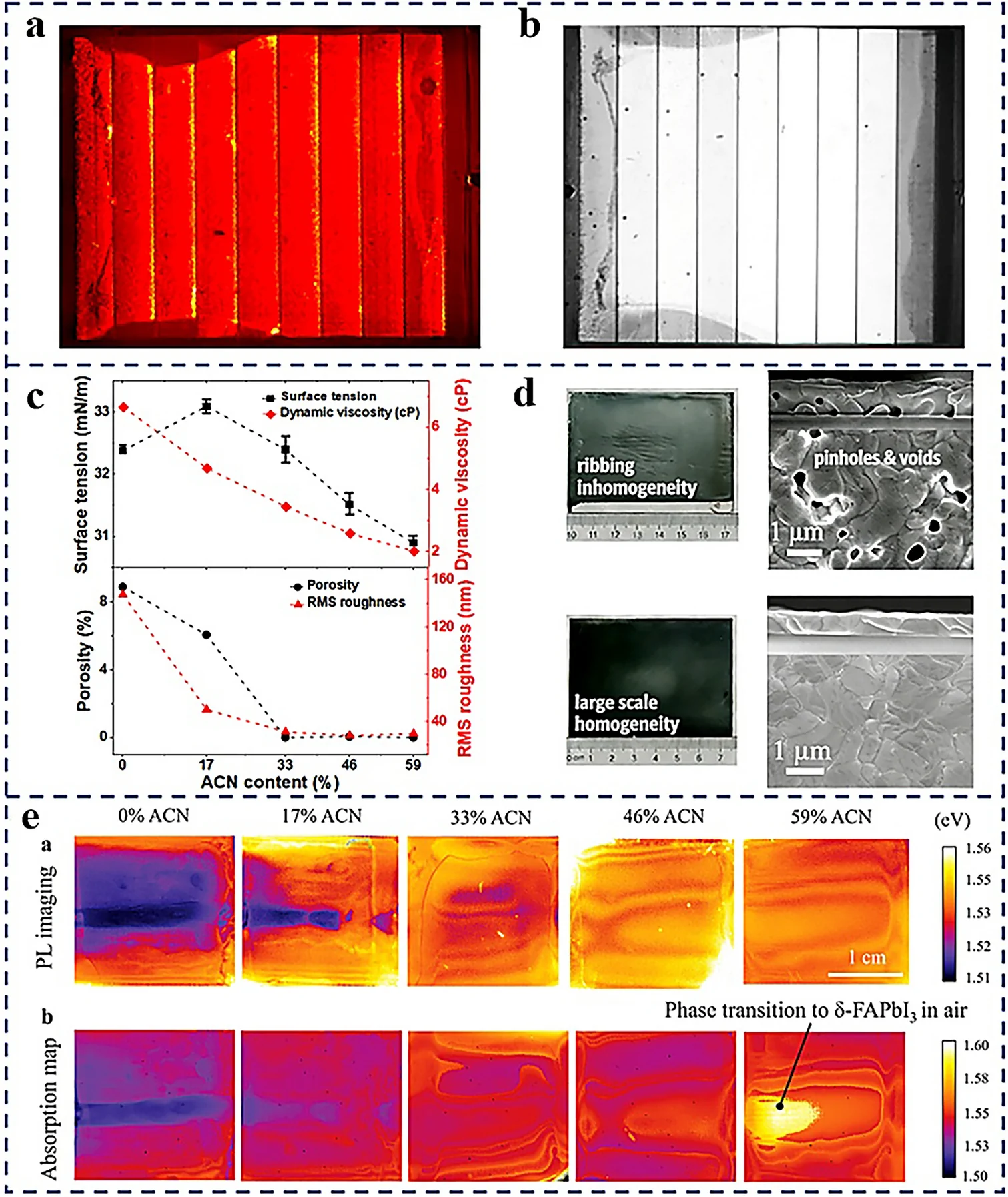
**a** and **b** Laser beam-induced current (LBIC) and electroluminescence (EL) mapping of a 3 × 3 in^2^ minimodule. Reproduced with permission from Ref. [146]. Copyright 2024, American Chemical Society. **c** Surface tension and dynamic viscosity of perovskite inks as well as the porosity and surface root-mean-square (RMS) roughness of the resulting thin films all vary with the ACN contents. **d** Images of annealed films (left column) and corresponding top/cross-sectional SEM views of the resulting perovskite films (right column). **e** PL peak position images of perovskite thin films. Reproduced with permission from Ref. [127]. Copyright 2023, John Wiley and Sons

To exploit high-boiling-point effects, N-cyclohexyl-2-pyrrolidone (CHP, boiling point: 206 °C) was incorporated, producing uniform films with enlarged grain sizes and enhanced crystallinity [71]. Extending this strategy, Rajakaruna et al. engineered a ternary solvent system comprising high-boiling-point (246 °C) 1,3-dimethyl-2-imidazolidinone (DMPU), DMF, and NMP. The DMPU component significantly extended the wet film processing window, facilitating perovskite films with superior uniformity via controlled nucleation (Fig. 9a, b) [146]. Notably, Sangale et al*.* introduced 10% 1,2-dichlorobenzene (DCB, 180 °C) into a DMSO-based triple-cation perovskite precursor ink, which substantially reduced the ink’s surface tension and viscosity while triggering the formation of locally supersaturated perovskite colloids. This strategy yielded a PCE of 20.61% on 0.1 cm^2^ active area devices [118]. Notwithstanding these advancements, a pivotal challenge endures: high-boiling-point solvents frequently become confined at the perovskite–substrate interface or within grain boundaries following thermal annealing. Such residual species give rise to morphological defects and voids at the interface—a phenomenon extensively corroborated in the literature to adversely affect device performance, chiefly by compromising charge transport dynamics and long-term operational stability [184–186].

#### Low-Boiling-Point Solvent Strategy

In parallel, the strategic deployment of low-boiling-point solvents represents a pivotal advancement in perovskite solvent engineering. Illustrating this, Abate et al*.* systematically investigated the composition-dependent effects of DMSO: acetonitrile (ACN, boiling point: 82 °C) solvent systems on CsPbI_2.77_Br_0.23_ precursor inks. Dynamic light scattering analysis revealed that increasing the ACN fraction markedly enhanced the abundance of sub-10-nm colloidal particles, resulting in perovskite films exhibiting highly uniform and pinhole-free morphology. Optimized formulations with DMSO: ACN at 4:1 (*v/v*) produced such films, which enabled slot-die-coated devices to record a maximum PCE of 19.05% [134]. A core rationale for this solvent engineering approach is the mitigation of coating defects, specifically the suppression of ribbing patterns stemming from capillary pressure imbalance. Extending this approach, research has shown that at an ACN volume fraction of 46%, the dynamic viscosity of the perovskite precursor solution is reduced to a critical value of 2.645 cP, thereby completely eliminating ribbing instabilities (Fig. 9c, d). Building on this principle, researchers have successfully fabricated pinhole-free FAPbI_3_ thin films with minimized surface roughness, attaining devices with a certified steady-state PCE of 22.30% [127]. Methodologically, non-contact PL imaging and absorption mapping permit precise thickness homogeneity assessment in halide perovskite absorber layers (Fig. 9e). These nondestructive techniques demonstrate dual utility: Functioning as in-process quality monitoring tools during thin-film fabrication and serving as high-throughput in-line metrology solutions for production-scale operations.

#### Coordinating Solvents and Crystallization Control

**Fig. 10:**
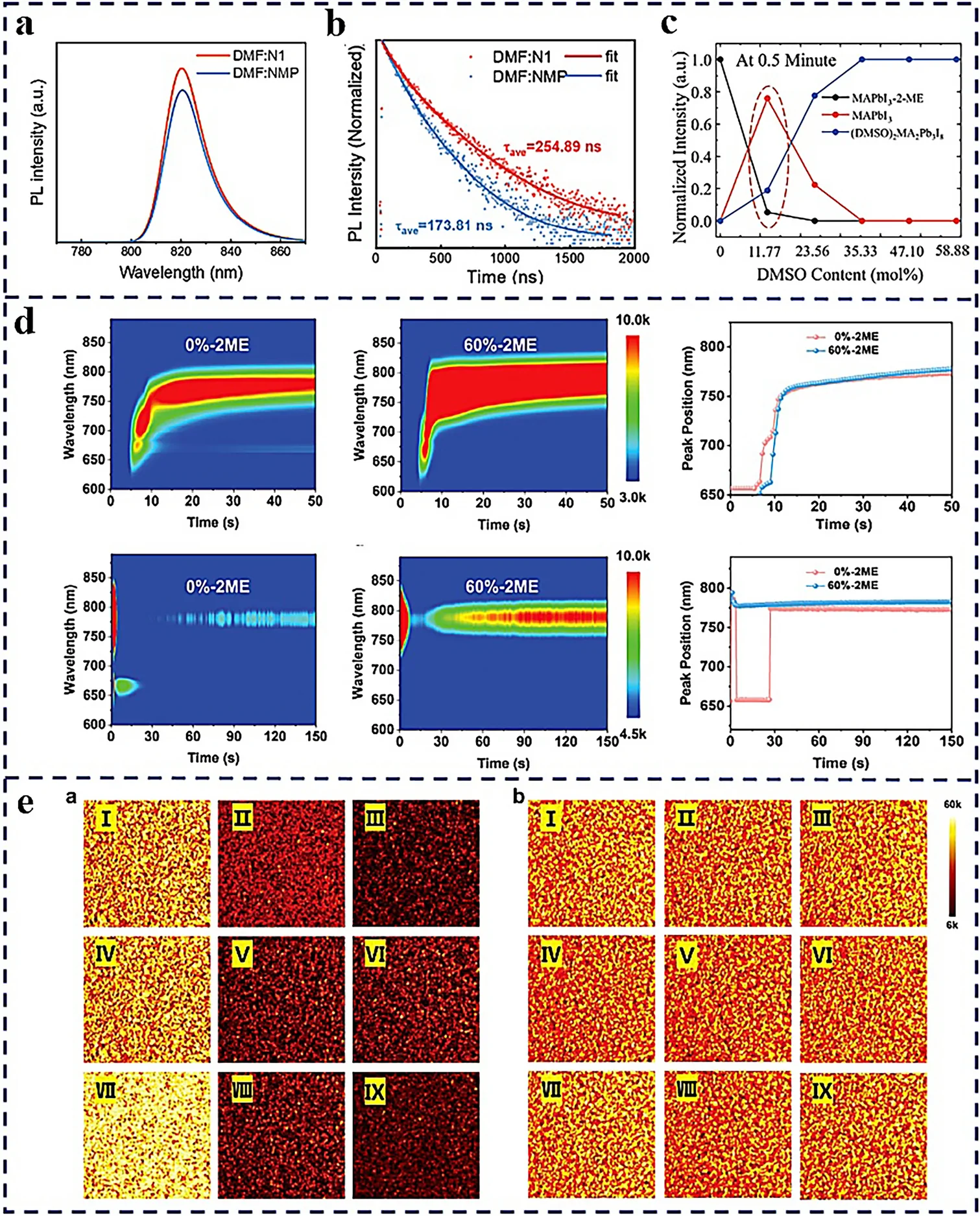
**a** and **b** Steady-state PL and TRPL spectra of the perovskite films using DMF:N1 or DMF:NMP as mixed solvent. Reproduced with permission from Ref. [145]. Copyright 2023, Elsevier. **c** MAPbI_3_-2-ME, MAPbI_3_ and (DMSO)_2_MA_2_Pb_3_I_8_ as function of DMSO content at 0.5 min of in situ GIWAXS pattern. Reproduced with permission from Ref. [94]. Copyright 2021, John Wiley and Sons. **d** in situ PL spectra and peak position evolution of 0%-2-ME versus 60%-2-ME samples during gas quenching and annealing. **e** 9-point PL mapping comparison: 0%-2-ME- vs. 60%-2-ME-based large-area perovskite films (5.0 × 5.0 cm^2^). Reproduced with permission from Ref. [25]. Copyright 2025, John Wiley and Sons

Shifting focus to solvent coordination effects, Lee et al*.* systematically compared DMSO, NMP, DMF, and γ-Butyrolactone (GBL) in perovskite crystallization kinetics. FT-IR analysis revealed S = O/C = O peak redshifts in DMSO/DMF/NMP, confirming their strong coordination with PbI_2_ [102]. Notably, partial substitution of DMF solvent with N-methyl-2-piperidone (N1) enhanced coordination with PbI_2_, forming stable PbI_2_-N1 complexes. These complexes mediated the template-assisted crystallization process between the lead halide framework and organic cations (e.g., MA⁺/FA⁺), thereby precisely regulating perovskite nucleation/growth kinetics. This mechanism produced high-quality films characterized by low defect–trap density and prolonged carrier lifetime (Fig. 10a, b). Benefiting from these advantages, large-area PSMs (5 × 5 cm^2^) delivered a champion PCE of 21.00% while retaining 80% initial efficiency after 1000 h illumination at 50% RH/30 °C (unencapsulated) [145]. In a complementary study, Huang et al*.* established a dual regulatory mechanism through the introduction of NMP cosolvent into the FACs-based DMF system. Crucially, NMP not only suppressed the formation of DMF-coordinated perovskite intermediate complexes but also stabilized an NMP-PbI_2_ adduct. This adduct underwent in situ reaction with pre-deposited FAI/CsI salts to directly crystallize into the α-phase perovskite, thereby circumventing the kinetically unfavorable solid–solid phase transformation via the δ-phase. Consequently, highly crystalline, uniform, large-area perovskite films were fabricated [143]. Furthermore, incorporating 11.77 vol% DMSO in 2-methoxyethanol (2-ME) suppressed the formation of the MAPbI_3_-2-ME intermediate phase while directly triggering MAPbI_3_ crystallization and concurrently generating a limited amount of the (DMSO)_2_MA_2_Pb_3_I_8_ intermediate phase (Fig. 10c), synergistically accelerating MAPbI_3_ formation kinetics during slot-die coating [94]. In a breakthrough exploitation of this synergy, Huang et al*.* introduced 2-ME as a partial substitute for DMF in the DMF/NMP (19:1, *v/v*) perovskite solvent system. in situPL characterization revealed that within the 60 vol% 2-ME system, the (FA-2-ME)PbI_3_ complex induced by 2-ME bypassed the intermediate δ-phase to directly trigger α-phase perovskite crystallization (Fig. 10d). Exploiting this mechanism, high-quality large-area (25 cm^2^) perovskite films with pinhole-free morphology, high uniformity, and phase purity were fabricated via slot-die coating (Fig. 10e). Consequently, small-area cells (0.062 cm^2^) and modules (15.64 cm^2^) posted PCEs of 24.20% and 21.84%, respectively [25]. Although indispensable in regulating perovskite crystallization and film morphology, conventional coordinative solvents such as DMF and DMSO pose substantial environmental and health risks due to their inherent toxicity and persistence [187–189]. These limitations not only conflict with green chemistry principles but also amplify operational challenges and economic costs in large-scale manufacturing and waste management.

#### Solvent Selection Crossroads: Balancing Performance, Toxicity, and Processability

As detailed in this section, solvent engineering is crucial for tuning perovskite ink properties (viscosity, surface tension, coordination ability) and film crystallization kinetics. Traditional, strongly coordinating solvents like DMF, DMSO, and NMP have dominated both laboratory-scale and early upscaling research due to their ability to efficiently form stable PbI_2_-solvent intermediates, providing a broad thermodynamic and kinetic window for precise crystallization control. However, their toxicity, the risk of residue formation due to high boiling points, and their conflict with the principles of green manufacturing present significant hurdles that must be overcome for large-scale production.

**Fig. 11:**
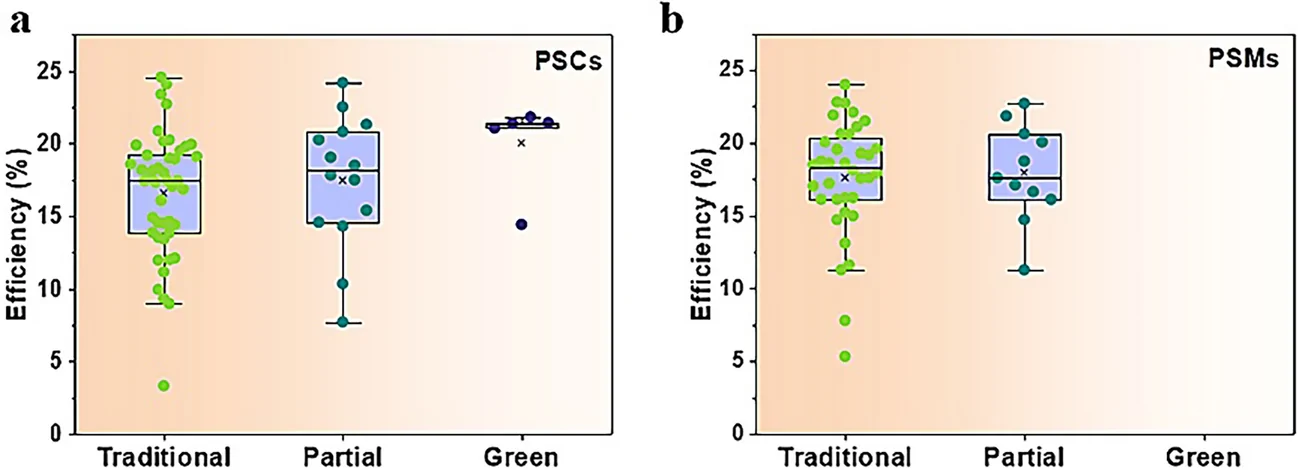
**a** Box plot distribution of efficiencies for slot-die-coated perovskite solar cells and** b** modules based on different solvent systems (2020–2025)

To quantitatively assess the current reliance on different solvent systems and their corresponding performance, we conducted a statistical analysis based on data from Tables 1 and 2 in this review, focusing on slot-die-coated PSCs/PSMs reported between 2020 and 2025 (Fig. 11a, b). We broadly categorized the solvent systems into three groups: (I) traditional strongly coordinating solvent-dominated systems (primarily DMF, DMSO, NMP, or their mixtures); (II) partial replacement/mixed solvent systems (e.g., introducing 2-ME, ACN as co-solvents but still containing traditional solvents); and (III) exploratory green/low-toxicity solvent systems (e.g., using GBL, 2-ME/ACN as the main solvent, or ionic liquids).

The analysis reveals a clear picture:

1) The Path of Record Efficiencies: All current slot-die-coated PSMs with efficiencies exceeding 22% (e.g., those cited in Table 2: [26, 27, 152, 157, 158]) rely heavily on traditional strongly coordinating solvents (Group I). These solvents, by forming stable intermediate phases, effectively widen the essential “processing window” for slot-die coating, which is key to producing large-area, uniform, high-quality films. This is the fundamental reason for their performance lead. 2) The Existence of an “Efficiency Gap”: Devices using partial replacement solvents (Group II) show median and champion efficiencies very close to those of traditional systems, demonstrating the feasibility of partially replacing toxic solvents through solvent engineering (e.g., using 2-ME, ACN to tune volatility and coordination). However, a significant gap exists for systems fully transitioning to exploratory green solvents (Group III), in terms of both reported champion efficiencies and median values (typically 20%-30% lower in relative efficiency compared to traditional systems). This constitutes a distinct “efficiency penalty” in the current transition toward green manufacturing. 3) The Root of the Gap: This gap is not insurmountable. Its origin lies in the weakened control over crystallization kinetics. Green solvents (e.g., certain alcohols, esters) often have weaker coordination ability or form less stable intermediates with perovskite precursors. This makes it difficult to precisely control nucleation and growth during the relatively slower drying process of slot-die coating, frequently resulting in porous, non-uniform film morphology and higher defect densities.

The above analysis indicates that current record-high performances are still achieved at the cost of using traditional toxic solvents. To achieve the unification of “green” and “high-performance” moving beyond simple solvent substitution is necessary; a coating-oriented ink system redesign is imperative. Future research should focus on:

1) Developing novel, strongly coordinating, low-toxicity/non-toxic solvent molecules to fundamentally replace DMF/DMSO. 2) Designing ingenious “solvent–additive” synergistic systems where green solvents serve as the main component, and functional additives (e.g., multidentate ligands) are used to precisely regulate the crystallization pathway, compensating for the weak coordination of the green solvent. 3) Optimizing drying processes tailored for green solvents (e.g., more precise gas quenching, vacuum flashing) to compensate for their narrower processing windows via external field assistance. 4) Establishing comprehensive evaluation criteria encompassing performance, environmental health and safety (EHS) assessment, life cycle analysis, and coating process window width to guide the field toward genuine sustainability. Bridging this “efficiency–environment–processability” trilemma is an essential step for slot-die-coated perovskite technology to evolve from a laboratory champion to an industrial titan.

### Additive Engineering

Beyond solvent engineering, additive engineering allows precise control over perovskite ink properties—specifically solvent volatility, rheological behavior, and coordination ability—to optimize film formation [190, 191]. Additives are broadly classified into two categories: (1) volatile additives (e.g., MACl, camphor and camphorquinone), which evaporate during thermal annealing and regulate crystallization kinetics through the formation of intermediate phases [192–195]; and (2) nonvolatile additives (e.g., BNCl, Piracetam, BnAm, [N4444][TFSI]), whose residual components segregate at grain boundaries or incorporate into the perovskite lattice as dopants via A-site substitution in ABX_3_ structures [196–199]. These nonvolatile additives typically perform three key functions simultaneously: (1) as defect passivators—passivating undercoordinated Pb^2+^/I^−^ sites through coordination/hydrogen bonding; (2) as ion migration suppressors—via hydrogen bonding-mediated halide anchoring; and (3) as hydrophobic barriers—blocking environmental moisture permeation. This multifunctional nature concurrently suppresses charge recombination, ion migration, and moisture degradation pathways, thereby maximizing PSCs/PSMs efficiency and stability.

#### Volatile Additives

Currently, MACl is the most widely used volatile additive in perovskite photovoltaics [200, 201]. For FA/Cs perovskite systems, its primary function involves strong coordination with PbI_2_ to form stable intermediate complexes. This effectively suppressed detrimental phases (φ-phase FAPbI_3_, PbI_2_-solvent intermediate phase), modulated nucleation/growth kinetics, and promoted dense, pinhole-free films with enlarged grains and reduced roughness. Such optimized morphology minimized non-radiative recombination, reaching a maximum PCE of 17.50% [95]. Analogously, Le et al*.* introduced trace MACl (in MA-based systems) or FACl (in CsFA-mixed systems) into precursor inks. These additives delayed nucleation via intermediate phase complexes (e.g., MAI–PbI_2_–MACl and FAI–PbI_2_–FACl), resulting in large-grained perovskite films [101]. In quasi-2D perovskites, MACl incorporation enhanced phase purity and vertical crystal orientation. This synergy substantially improved charge transport in R2R slot-die-coated devices, achieving a high PCE of 9.30%, and later enhanced to 12.50% [83, 87]. Expanding beyond MACl, chloride-based additives such as NH_4_Cl and HCl have been rigorously validated. During crystallization, NH_4_Cl formed NH_4_PbI_3_ intermediate phases that templated the epitaxial growth of MAPbI_3_ crystals, inducing preferentially oriented growth and enhancing film crystallinity. Concomitantly, grain enlargement suppressed non-radiative recombination at grain boundaries, elevating PCE to 15.57% [80]. For slot-die-coated MAPbI_3_, HCl addition increased grain size from 300 nm to ≈ 1 μm, yielding PCEs of 14.70% (rigid substrates) and 12.80% (flexible substrates) [85]. It should be noted, however, that although volatile chloride additives (e.g., MACl) demonstrate notable advantages in modulating perovskite crystallization, their practical implementation faces several limitations, such as the asynchronous volatilization of MA^+^ and Cl^−^, which can lead to additive residues and consequent adverse effects on the long-term operational stability of the devices [202–204].

Replacement of PbI_2_ with Pb(OAc)_2_ led to rapid perovskite crystallization via swift volatilization of byproduct CH_3_NH_3_OCOCH_3_ (MAAc), with low annealing temperatures (< 100 °C) being ideal for flexible devices [74]. However, Pb(OAc)_2_ produced submicron-sized grains (< 300 nm), limiting PCE to < 10%. To overcome this limitation, Lee et al*.* incorporated 20% PbCl_2_ into Pb(OAc)_2_-based inks. During annealing, PbCl_2_ decomposition released Cl^−^ containing vapors that passivated grain boundaries and facilitated Ostwald ripening—thereby alleviating void formation at the bottom interface while increasing grain size. This strategy produced devices with a PCE of 13.30% [78]. The long-term stability concerns associated with residual halides, as mentioned previously, warrant further investigation for these systems as well.

#### Nonvolatile Additives

**Fig. 12:**
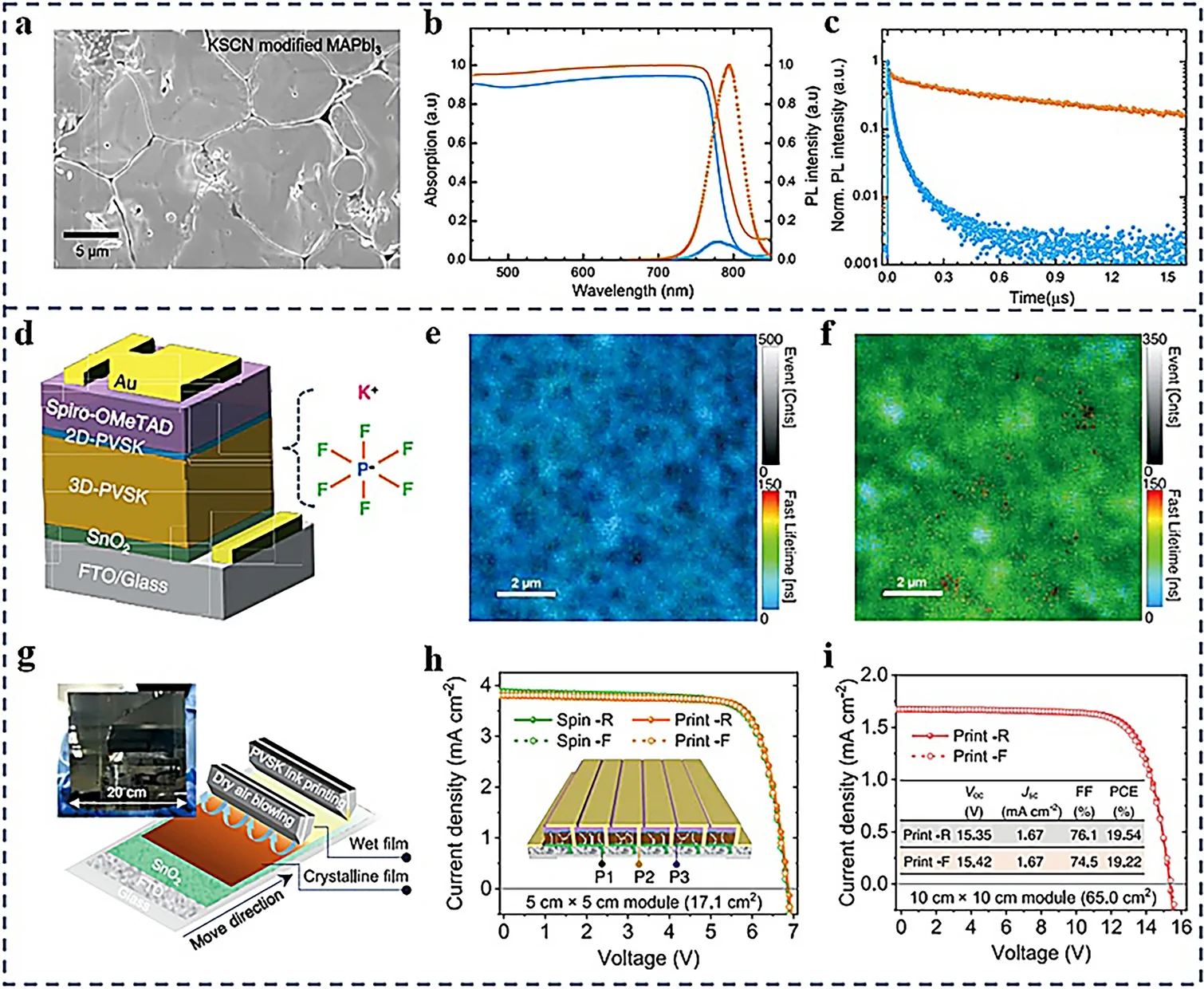
**a** Top view SEM image of KSCN-modified MAPbI_3_ films. **b** Absorptance and steady-state PL spectra, **c** TRPL spectra of pristine and KSCN-modified MAPbI_3_ films. Reproduced with permission from Ref. [98]. Copyright 2021, John Wiley and Sons. **d** Structural schematic of PSCs. **e** and **f** Time-resolved confocal PL lifetime maps of 3D/2D and 3D/2D KPF_6_ perovskite films, respectively. **g** Schematic illustration of the slot-die printing of perovskite films. **h** *J–V* curves of the 5-cm-by-5-cm minimodules. **i** *J–V* curves of the 10-cm-by-10-cm minimodules. Reproduced with permission from Ref. [143]. Copyright 2021, American Association for the Advancement of Science

Potassium salts were extensively utilized in slot-die-coated PSCs. Exemplified by potassium thiocyanate (KSCN), thiocyanate anions (SCN^−^) exhibited high binding affinity toward Pb^2+^, effectively suppressing nucleation while promoting perovskite grain growth (average size ≈ 11 μm, Fig. 12a). Concurrently, potassium ions (K^+^) migrated to grain boundaries to passivate uncoordinated iodide species (I^−^), thereby reducing trap-state density. These synergistic effects significantly enhanced optoelectronic properties (Fig. 12b, c), enabling the fabricated PSCs to record a maximum PCE of 21.38% with negligible hysteresis [98]. Similarly, large-sized and effectively defect-healed FAPbI_3_ films via KSCN additive were reported by other researchers [117]. Beyond small-molecule additives, crystalline templating strategies also leverage potassium salts. For example, implementation of a KPb_2_Br_5_ seed-assisted crystallization strategy to template grain growth enabled PSMs (active area of 57.50 cm^2^) to demonstrate a high efficiency of 16.22%. Owing to improved crystallinity and grain boundary passivation, the KPb_2_Br_5_-seeded PSMs maintained 82% of their initial PCE after 4800 h of ambient storage at 30% relative humidity without encapsulation [108]. Furthermore, extending the concept of multifunctional additive engineering, Bu et al*.* introduced potassium hexafluorophosphate (KPF_6_) into the precursor solution of CsFAMA triple-cation perovskite (Cs_0.05_(FA_0.85_MA_0.15_)_0.95_Pb(I_0.85_Br_0.15_)_3_). This approach achieved dual-functional defect passivation through the synergistic effects of K^+^ and PF_6_^−^ ions derived from its dissociation. Specifically, K^+^ ions effectively passivated A-site vacancies within the perovskite lattice, while PF_6_^−^ ions passivated interface/grain boundary defects via hydrogen bonding with organic cations (e.g., FA^+^, MA^+^) and significantly suppressed ion migration across the perovskite layer. This synergistic passivation strategy markedly enhanced the optoelectronic properties of perovskite films (Fig. 12d-f). Experimental results demonstrated that minimodules fabricated via slot-die coating achieved PCEs of 20.42% and 19.54% on active areas of 17.10 and 65.00 cm^2^ (Fig. 12g-i), respectively [143]. This work highlights the exceptional scalability of the KPF_6_ additive strategy for industrial PSCs production, providing critical technical support for the efficient and reproducible fabrication of large-area perovskite photovoltaic devices.

Despite their remarkable efficacy in boosting the performance of perovskite solar cells, potassium salt additives face formidable challenges that hinder their practical deployment. The pronounced ionic radius mismatch between K^+^ (~ 1.38 Å) and the prevalent A-site cations (MA^+^, ~ 1.80 Å; FA^+^, ~ 2.2 Å) induces severe lattice strain upon excessive incorporation, triggering phase segregation and the formation of deleterious non-perovskite phases (e.g., KPbI_3_). These secondary phases act as charge recombination centers, compromising both device efficiency and operational stability. Furthermore, the migration dynamics of K^+^ ions under operational stressors (electric field, light, heat) and their ensuing accumulation at critical interfaces remain poorly elucidated, posing risks of interfacial barrier formation and rapid performance degradation. The prevailing narrative of “synergistic passivation” is primarily built on macroscale properties and conventional characterization (SEM, XRD, PL). However, a fundamental understanding of the atomistic mechanisms—such as the precise lattice site occupancy of K^+^ and its role in modulating hydrogen bonding networks—lags behind, crippled by a scarcity of in situ/operando studies and rigorous theoretical modeling. Pioneering research efforts must therefore prioritize pinpointing the optimal concentration window, assessing compatibility within multicomponent perovskite systems, and employing advanced in situ probes to visualize ion migration, thereby laying a robust foundation for industrial translation.

#### Multifunctional Synergistic Effects of Additives

**Fig. 13:**
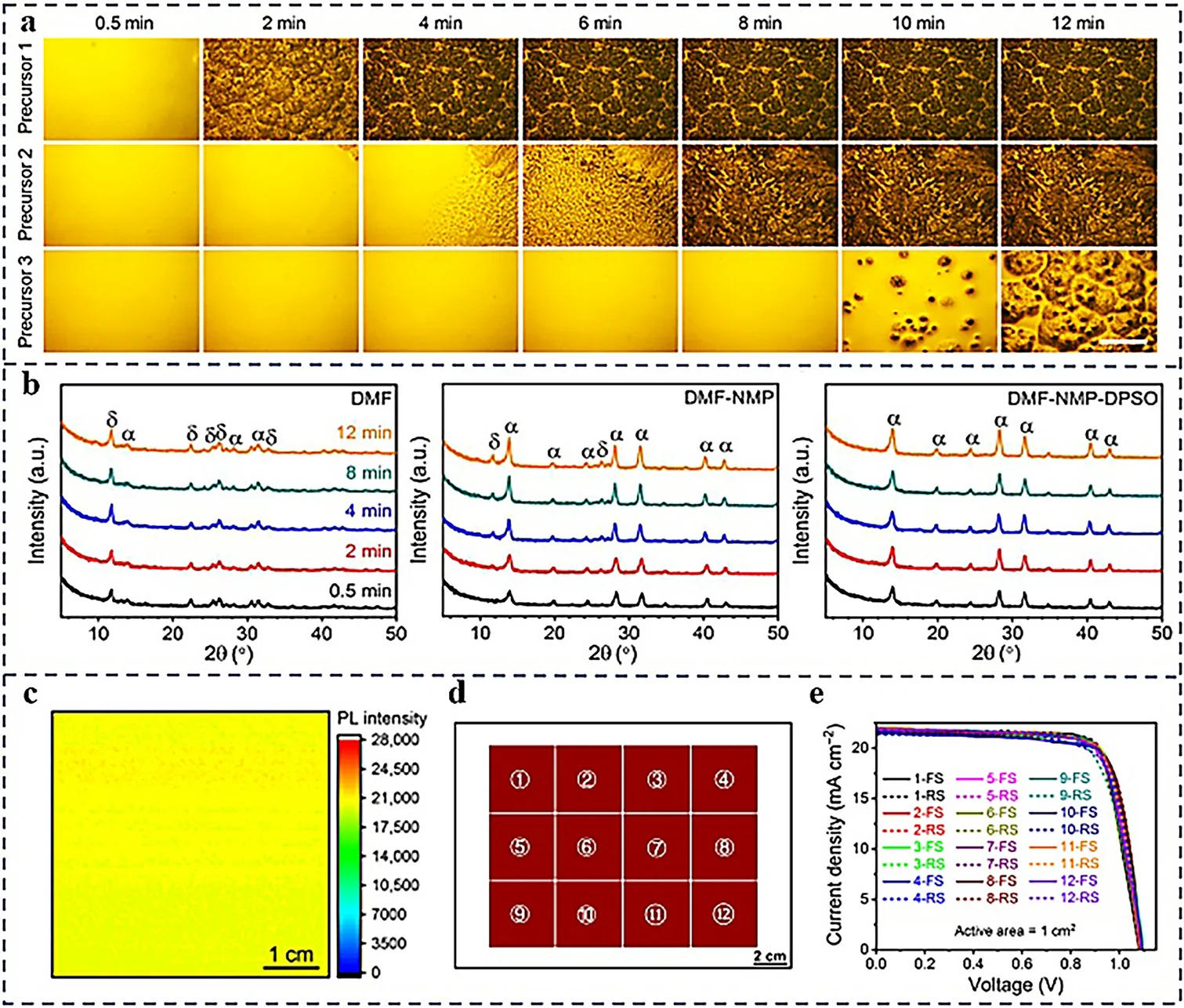
**a** Semi-in situ optical microscopy observation of morphology evolution for perovskite precursor films in different solvent systems (scale bar: 100 μm). Precursors 1, 2, and 3 correspond to DMF, DMF–NMP, and DMF–NMP–DPSO, respectively. **b** XRD patterns of three precursor wet films after immersion in anti-solvent for 2 min at different time stages (without annealing). **c** PL mapping image. **d** Schematic of dividing a large-area perovskite film into 12 pieces (4 cm × 4 cm each). **e** *J–V* curves of 12 PSCs. Reproduced with permission from Ref. [43]. Copyright 2021, American Association for the Advancement of Science

Organic additives with tailored functional groups have been demonstrated to enable multifunctional modulation of slot-die-coated perovskite films, contributing to enhanced moisture resistance, optimized precursor solution rheology, governed crystallization kinetics, inhibited colloidal aggregation, improved interface modification, and promoted defect passivation [205, 206]. Regarding moisture protection, poly(ethylene oxide) (PEO) inhibited hydrate formation under high humidity via hydrogen bonding with MA^+^ cations during coating [86], while cornstarch polymers anchored MA^+^ to produce moisture-resistant films [92]. For ink processability, the L-α-phosphatidylcholine (LP) additive modulated the rheology of the precursor solution, which not only significantly enhanced the wetting performance of perovskite inks on hydrophobic PTAA substrates but also effectively suppressed surfactant-driven Marangoni effects occurring during crystal growth [89, 93]. To regulate crystallization kinetics, diphenyl sulfoxide (DPSO) served as a strong Lewis acid additive. Through the formation of a stable PbI_2_–DPSO intermediate complex, DPSO elevated the nucleation barrier, widening the processing window (Fig. 13a). Critically, it suppressed δ-FAPbI_3_ impurity formation while directly inducing α-FAPbI_3_ phase nucleation (Fig. 13b), ultimately producing high-quality FA_x_Cs_1-x_-based films. This refined crystallization process resulted in uniform PL intensity distribution across the film, combined with highly consistent performance of 12 independent devices (Fig. 13c-e), collectively confirmed exceptional large-area uniformity. This strategy delivered parallel interconnected modules with a certified quasi-steady-state efficiency of 16.63% (active area: 20.77 cm^2^) [43]. In the realm of modulating colloidal aggregation, Zhang et al. introduced N-cyano-4-methyl-N-phenylbenzenesulfonamide (CMPS) into the perovskite precursor. Employing its multifunctional moieties—including benzene rings, cyano, and sulfonyl groups—CMPS effectively suppressed colloidal aggregation, thereby facilitating the fabrication of high-quality, large-area perovskite films under ambient conditions via the slot-die coating technique [140].

**Fig. 14:**
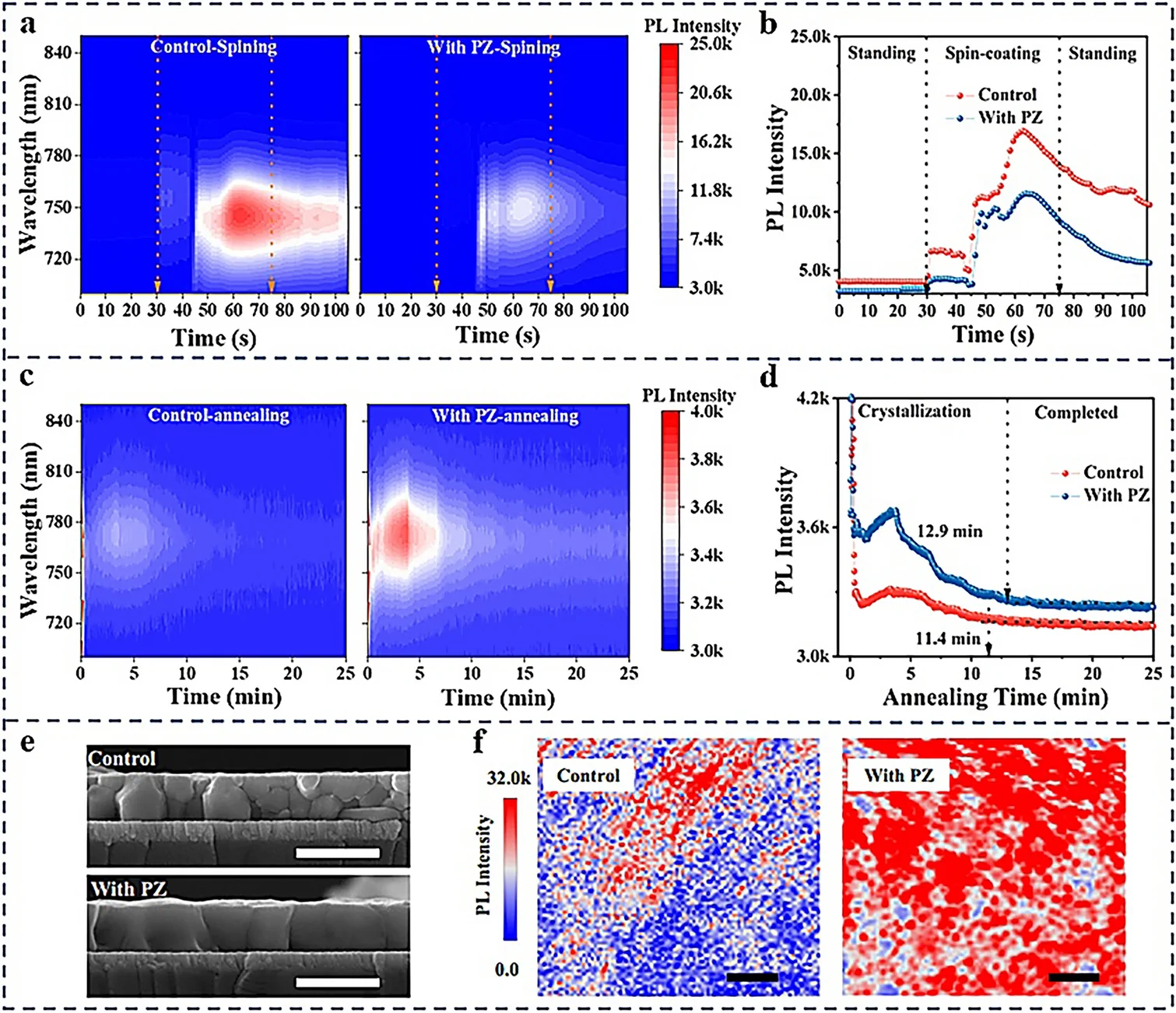
**a** Real-time PL spectra of perovskite wet films recorded during the spin coating process. **b** Real-time PL intensity at 760 nm of perovskite wet films during spin coating process. **c** Real-time PL spectra of perovskite semi-wet films recorded during the annealing process. **d** Real-time PL intensity at 760 nm of semi-wet films during annealing. **e** Cross-sectional SEM images of perovskite films. Scale bars, 1 µm. **f** PL mapping images of perovskite films. Scale bars, 4 µm. Reproduced with permission from Ref. [139]. Copyright 2025, Springer Nature

Similarly, Yuan et al*.* introduced pyrazolidine (PZ) into the perovskite precursor solution. By suppressing colloidal aggregation and retarding crystallization kinetics, PZ ensured a spatially constant growth rate throughout the film (Fig. 14a-e). PL mapping further revealed that PZ-treated films exhibit significantly enhanced uniformity in luminescence intensity compared to control samples (Fig. 14f), unambiguously verifying the optimization effect on film homogeneity [139]. For interface modification and beyond, Zang et al. added morpholine-4-formamidinium chloride (M4CH) into the perovskite precursor solution. Owing to its strongest adsorption energy on the (001) crystallographic plane of perovskite, M4CH induced vertically oriented growth of the film. Simultaneously, at the buried interface, M4CH accumulated to form a positive dipole layer, which optimized energy-level alignment and thus significantly enhanced carrier extraction and transport properties. Based on this strategy, an excellent performance of 18.54% (certified 18.48%) was obtained on a 642 cm^2^ module [154]. Beyond crystallization control, defect passivation is equally critical for high-performance devices. Compounds such as 2-(2,3,4,5,6-pentafluorophenyl) ethylammonium iodide (FEAI) [105], diglycolic acid (DA) [100], and 2-hydroxyethyl acrylate (HEA) [103] effectively passivated undercoordinated Pb^2+^ and halide vacancies via coordination bonds, allowing the fabrication of low-defect-density perovskite photovoltaics with enhanced PCEs. While organic additives provide multifunctional benefits, their application raises concerns regarding their intrinsic stability, which remains a critical research challenge. Many organic additives are susceptible to chemical degradation or molecular structural changes under various environmental stresses, including light, heat, and electric fields. The decomposition of additives not only compromises their original functionality but also generates byproducts that can act as new degradation initiators, thereby accelerating the performance decay of perovskite devices.

For nonvolatile organic additives, the overarching design principle should transcend single-point defect passivation. The ideal additive should multitask: it must simultaneously regulate ink rheology for stable coating, modulate crystallization kinetics for uniform film formation, passivate bulk and interface defects, and enhance intrinsic stability against environmental stressors. The long-term chemical stability of these organic molecules themselves under operational conditions is a critical but often overlooked criterion that demands systematic investigation.

### Interface Engineering

Interface engineering—particularly at perovskite/carrier-selective layer (CSL) junctions—is paramount for simultaneously enhancing photovoltaic performance and operational stability [207, 208]. In such heterojunctions, suboptimal energy-level alignment is widely recognized as the dominant factor in photovoltage losses [209, 210], driven by mechanisms such as Fermi-level pinning or insufficient interface dipole. In inverted (p–i–n) architectures, fullerene derivatives (e.g., PC_61_BM, C_60_) remain the dominant electron transport layer (ETL) materials due to their excellent electron affinity and energy-level alignment. Recent comprehensive reviews have summarized significant advances in optimizing fullerene ETLs through strategies such as defect passivation, energy-level tuning, and suppression of self-aggregation, which are crucial for high-performance devices [211]. Beyond energy alignment, undercoordinated species (e.g., I^−^, Pb^2+^) at surfaces and grain boundaries act as non-radiative recombination centers, severely compromising carrier diffusion length; moreover, these defects establish ion migration pathways and induce halide phase segregation kinetics, leading to synergistic deterioration of device efficiency and stability [212, 213]. Strategic interlayer design thus aims to passivate such defects via targeted chemical bonding [214, 215]. Concurrently, the physicochemical properties of substrates—including wettability and surface functional groups—govern perovskite crystallization kinetics, necessitating precise interface tuning [62, 112]. Beyond bulk interface control, molecularly tailored capping layers (e.g., 2D perovskites or cross-linkable polymers) suppress environmental degradation and ion migration by elevating activation barriers, thereby extending operational stability against light/heat stressors [216, 217]. It is worth noting that the most efficient interface engineering strategies often aim to synergistically integrate the aforementioned multiple functions. A landmark advancement in this regard is the adoption of an Nd@C_82_–polymer coupling layer as an electron-selective interface. This design innovatively combines the ultrafast electron extraction and favorable energy-level alignment enabled by the endohedral metallofullerene, the in situ encapsulation effect analogous to a capping layer provided by the polymer for suppressing ion migration and environmental degradation, as well as the inherent defect passivation capability of the coupled interface itself. As a result, the device achieves industry-leading performance in both power conversion efficiency (26.78%) and operational stability under harsh conditions (82% retention over 2500 h), exemplifying the decisive role of function-integrated interface engineering in advancing the industrialization of perovskite photovoltaics [218].

#### Bottom Interface Engineering

**Fig. 15:**
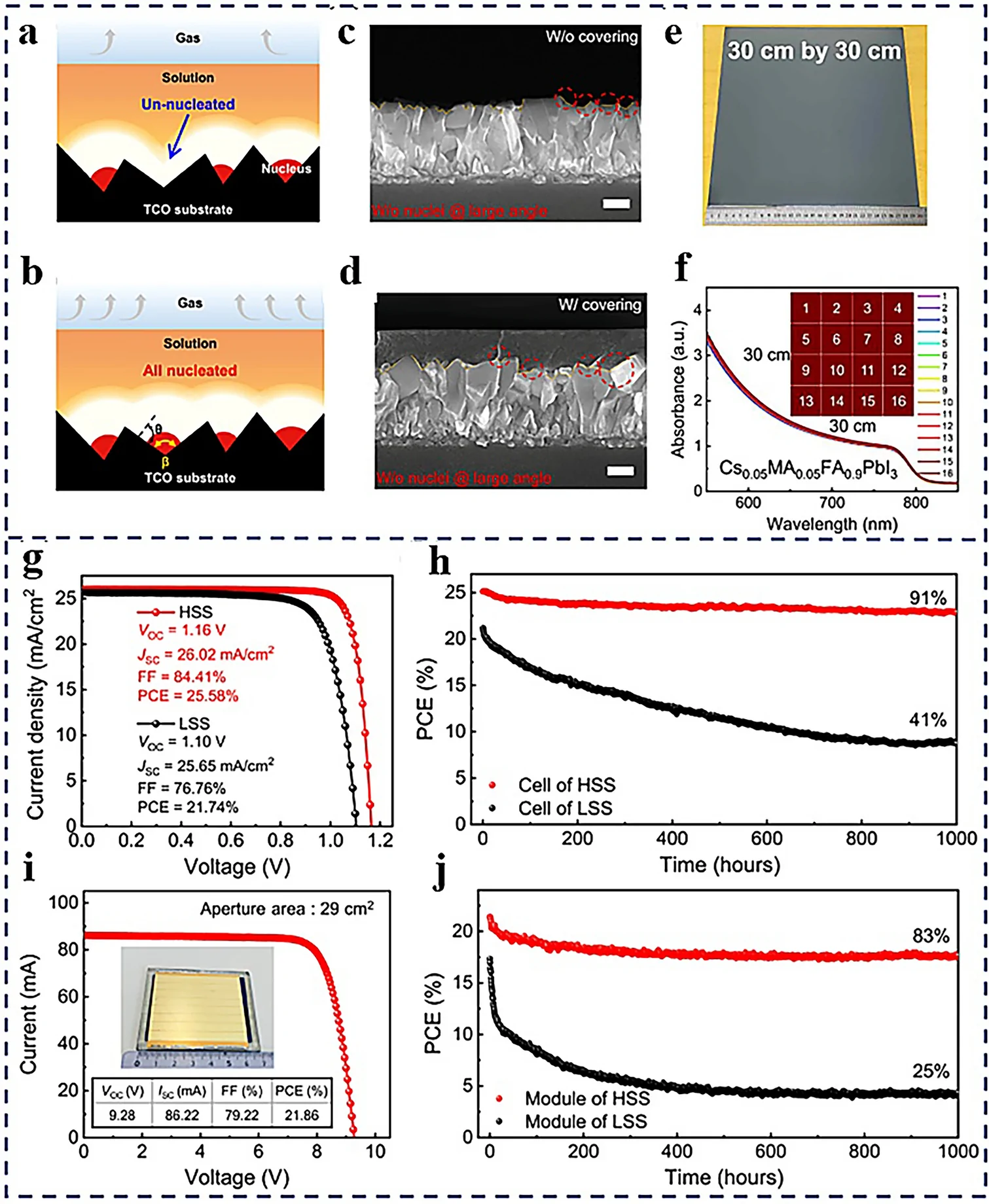
**a** and** b** Comparative schematic illustrating the dependence of heterogeneous nucleation on supersaturation: suppression at large-angle concavities under low supersaturation (LSS) versus complete nucleation coverage under high supersaturation (HSS). **c** and **d** Preferential nucleation at concavities: Cross-sectional SEM of perovskite films on rough FTO without/with a capping layer. Scale bars: 200 nm. **e** Perovskite films. **f** UV–Vis absorption spectra obtained from 16 different locations. **g** *J–V* curves of champion PSCs. **i** *I–V* curve of champion PSM. **h** and** j** Operationalstability of the encapsulated PSCs and PSMs measured at MPPT under a continuous AM1.5G illumination in air. Reproduced with permission from Ref. [44]. Copyright 2024, American Association for the Advancement of Science

Interface engineering at the bottom substrate critically governs perovskite film formation and device performance. As demonstrated on metal oxide interfaces, corona treatment rapidly removed contaminants and enhanced ZnO films’ wettability [70]. However, tin dioxide (SnO_2_) electron transport layers face intractable challenges: inherent surface defects and irreversible agglomeration. To overcome these, Sun et al*.* strategically incorporated diethyl 2-chloromalonate (DCMA) during the synthesis of SnO_2_ nanoparticles. The grafting reaction promoted the in situ formation of a homogeneous shell layer, which effectively passivated oxygen vacancies through O–Sn coordination while simultaneously inhibiting particle agglomeration. Optimized energy-level alignment further enhanced charge extraction and transport kinetics, resulting in a synergistic enhancement in *V*_*OC*_ and fill factor (FF) [150]. In a separate approach, to suppress nucleation competition at concave geometries on rough-textured SnO_2_ substrates, Zhang et al*.* proposed a supersaturation regulation strategy. By implementing accelerated drying, this approach induced a high-supersaturation state, thereby homogenizing nucleation kinetics across heterogeneous concave sites and suppressing the formation of pinhole defects and interfacial voids (Fig. 15a-d). As a result, large-area uniform perovskite films were fabricated (Fig. 15e, f), enabling devices to achieve high PCEs of 25.58% (aperture area 0.06 cm^2^) and 21.86% (aperture area 29 cm^2^), along with exceptional operational stability under continuous illumination (Fig. 15g-j) [44]. For TiO_2_-based interfaces, potassium-doped graphene oxide (GO-K) employed as an interlayer optimized band alignment and effectively suppressed interfacial charge recombination losses [96].

**Fig. 16:**
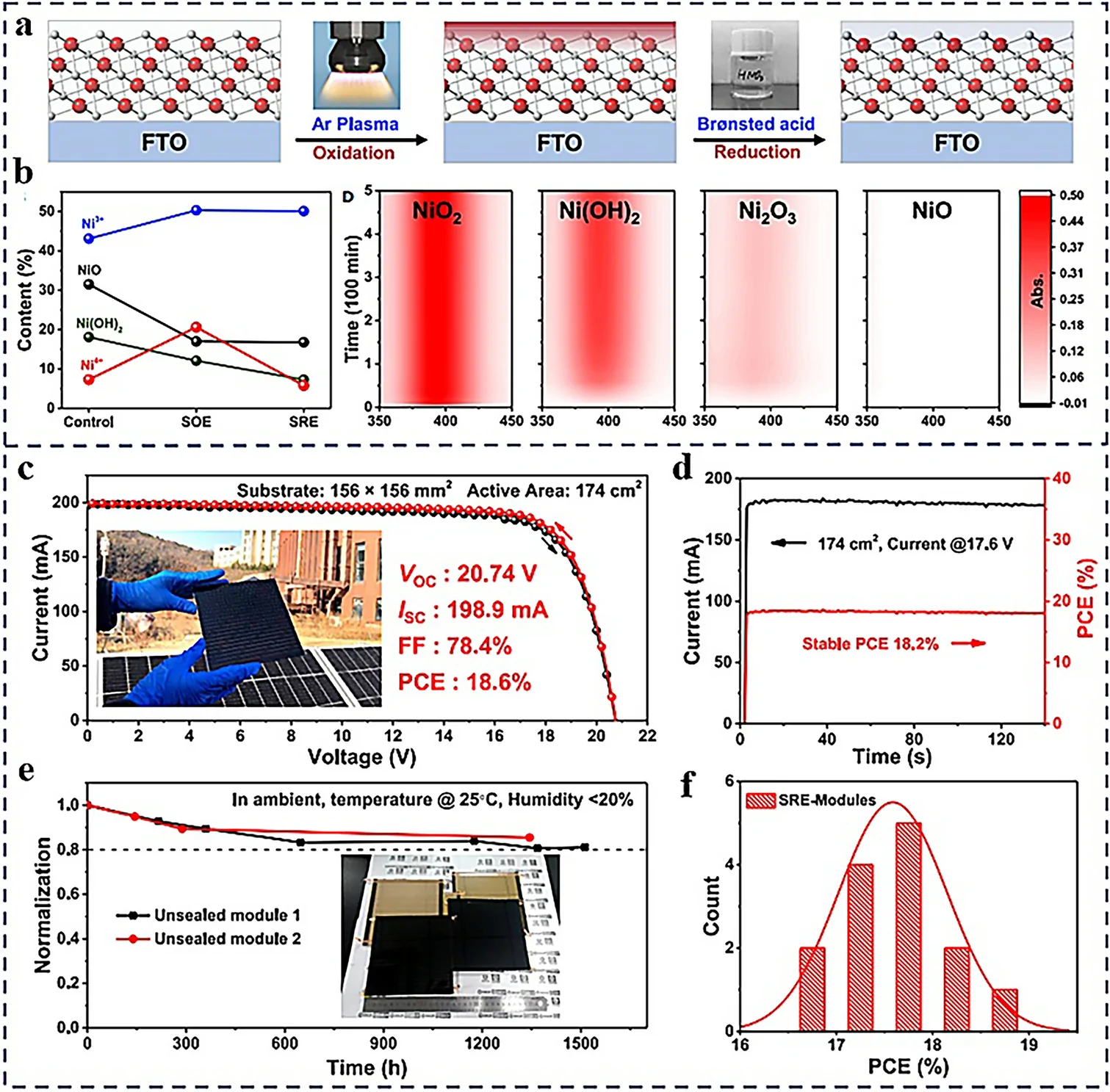
**a** Schematic illustration showing the process of SRE. **b** The variation of Ni species caused by SRE and UV–Vis absorption spectra of solutions of NiO_2_/Ni(OH)_2_/Ni_2_O_3_/NiO powder dissolved in 3 M HNO_3_. **c** *J–V* curves of the champion module. **d** Steady-state power output of the champion module. **e** Long-term stability of two modules aged in the ambient environment. **f** PCE distribution of 14 modules. Reproduced with permission from Ref. [112]. Copyright 2021, Cell Press

At the NiO_x_ interface, Ni^3+^ species engendered by surface redox engineering (SRE) concomitantly enhanced the electrical conductivity of the NiO_x_ film (Fig. 16a, b) and elevated its surface energy. This synergistic effect significantly improved the crystallinity and surface coverage of perovskite layers deposited on NiO_x_ substrates. By adopting this strategy, large-area, pinhole-free, and highly uniform perovskite films were fabricated on NiO_x_ using slot-die coating. Rigid and flexible PSCs attained impressive PCEs of 23.40% and 21.30%, respectively. Furthermore, a perovskite submodule with a designated aperture area of 156 × 156 mm^2^ was realized, attaining a PCE of 18.60% while demonstrating excellent operational stability upon aging in the dark for 1500 h (Fig. 16c-f) [112]. Additionally, treatment of NiO_x_ with L-ascorbic acid (L-AA) utilized protons derived from enolic hydroxyl ionization to suppress organic cation deprotonation, thereby improving precursor solution stability. Concurrently, hydroxyl/carbonyl coordination to Pb^2+^ directed crystallization kinetics and passivated undercoordinated Pb defects. This strategy yielded 19.17%-efficient modules (57.30 cm^2^ active area) [149]. Beyond metal oxides, molecular engineering of organic transport layers has proven equally effective. Incorporating guanidinium iodide (GAI) into Poly(3,4-ethylenedioxythiophene) (PEDOT:PSS) established hydrogen bonding with halide frameworks, which enhanced perovskite crystallinity, reduced PbI_2_ impurities, and optimized energy-level alignment for carrier extraction [99]. Further gains arose from 3-aminopropanoic acid self-assembled monolayers (C3-SAM) on PEDOT:PSS [65]. Advancing the molecular design of hole transport materials themselves, particularly for the critical buried interface in inverted (p–i–n) architectures, represents a pivotal direction. A notable example is the work by Zhao et al., who incorporated self-assembled molecules (SAMs) into a tris(pentafluorophenyl)borane (BCF) matrix. This approach effectively suppressed the inherent aggregation and crystallization tendencies of SAMs, resulting in the formation of an amorphous and uniformly covered charge transport layer. The strategy concurrently enhanced the wettability of the perovskite precursor solution on the hole transport layers (HTL), improved the final interfacial contact quality, reduced non-radiative recombination and interfacial stress, and ultimately yielded a PCE of 26.11% [156]. This work exemplifies the profound potential of intrinsic material engineering for achieving high-performance buried interfaces.

Even with remarkable research advances, the field of buried interface engineering continues to face significant challenges. A critical issue lies in the intrinsic conflict between chemical stability and electrical functionality: many high-performance interfacial modifiers, such as certain organic molecules, exhibit questionable long-term chemical stability under persistent light soaking and thermal stress. These materials may themselves evolve into degradation initiators, ultimately compromising device longevity. A more fundamental challenge originates from energy-level mismatch under dynamic operating conditions. The energy levels of perovskite materials undergo substantial shifts in response to illumination and electrical bias—a phenomenon known as light-induced band shifting—while those of charge transport layers remain largely static. Most current studies rely on static energy-level measurements and alignment strategies; however, the understanding of dynamic energy-level alignment under realistic operating conditions remains in its infancy, representing a critical bottleneck hindering further improvements in device performance and stability. Future interface design must account for this dynamic behavior to create more robust heterojunctions.

#### Top Interface and 2D Capping Layer Engineering

**Fig. 17:**
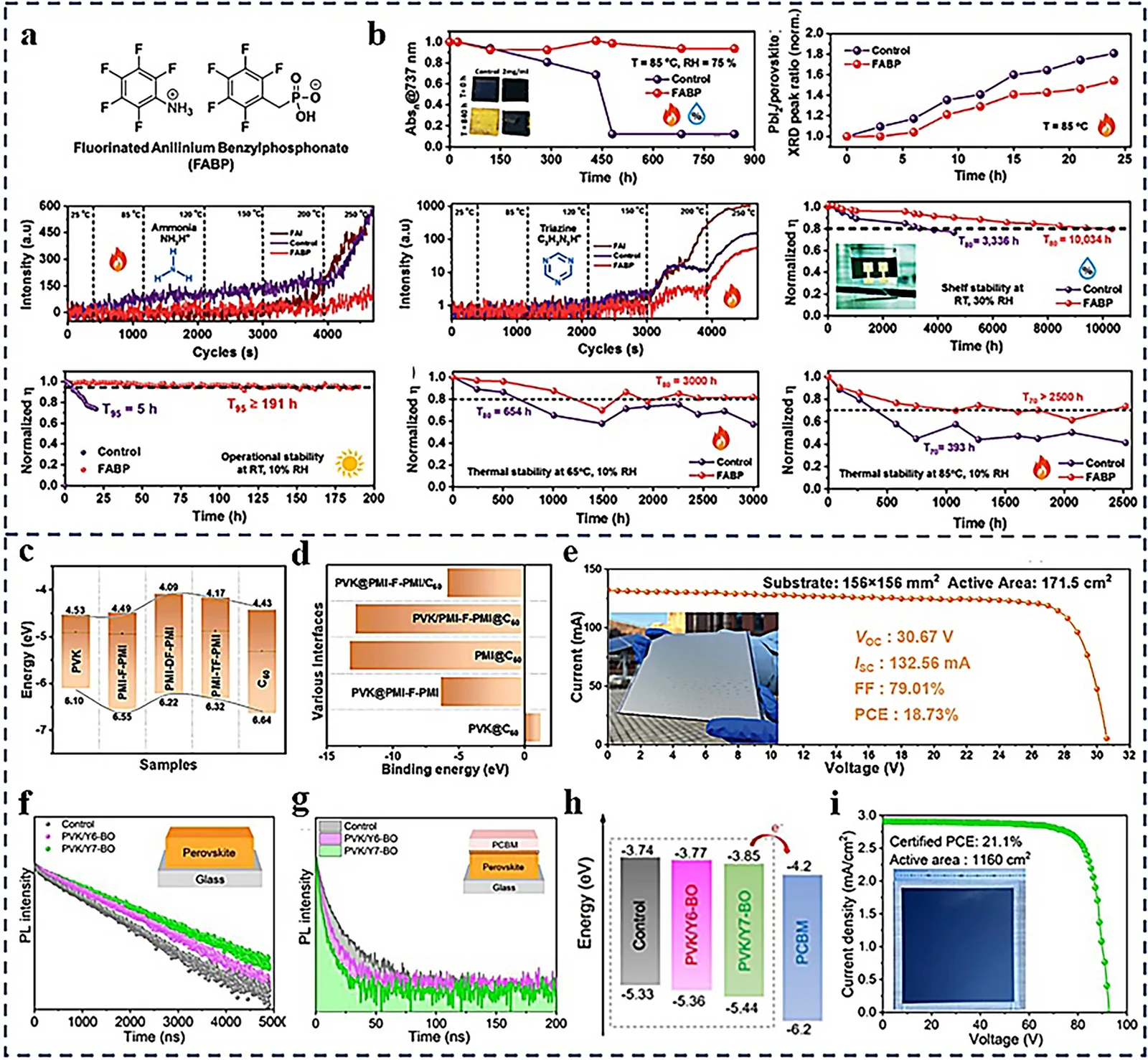
**a** Molecular structure of FABP. **b** Stability comparison of control and FABP-treated samples. Reproduced with permission from Ref. [123]. Copyright 2023, John Wiley and Sons. **c** Energy band structure of various films. **d** Binding energy of various systems. **e** *I–V* curve of the champion PSMs. Reproduced with permission from Ref. [148]. Copyright 2024, John Wiley and Sons. **f** TRPL spectra. **g** TRPL spectra. **h** Energy-level alignment of Control, PVK/Y6-BO and PVK/Y7-BO films. **i** *J–V* curve of i-PSMs with areas of 1160 cm^2^. Inset: PCEs and photographs of the device. Reproduced with permission from Ref. [151]. Copyright 2024, John Wiley and Sons

The introduction of organic molecules with tailored functional groups enables multifunctional synergistic modulation at the top interface of slot-die-coated perovskite films, thereby achieving surface defect passivation, precisely tailored energy-level alignment, enhanced interfacial energetics, and ultimately improved environmental stability [219, 220]. Research has demonstrated that ionic liquids (e.g., [M_4_N]^+^BF_4_^−^) utilized BF_4_^−^ anions to form strong ionic bonds (binding energy: 7.34 eV) with undercoordinated lead, reducing the surface Pb^0^/(Pb^0^ + Pb^2+^) ratio from 8% to < 1%. This concurrently suppressed deep-level trap states induced by iodine vacancies and inhibited gold electrode diffusion—an effect attributed to the strengthened interface barrier resulting from ionic bonding—elevating small-area device efficiency to 22.73% while achieving a stabilized efficiency of 18.60% for 40 × 40 mm^2^ modules [91]. Expanding this strategy, engineered artificial peptides (e.g., sulfonyl-γ-AA peptides) harnessed multifunctional carbonyl/carboxyl/sulfonyl groups to cooperatively heal dual-charge defects (e.g., uncoordinated Pb^2+^ ions and halide vacancies) through Lewis acid–base coordination and hydrogen bonding. Simultaneously, the synergistic effect of F-GLU-S’s hydrophobicity and defect healing capability effectively impeded moisture and oxygen penetration [111, 126]. Furthermore, Rana et al*.* employed hydrophobic fluorinated anilinium benzylphosphonate (FABP) (Fig. 17a) to passivate the top interface of FACs-based perovskite films. This passivator established a “molecular lock” mechanism via dual-anchoring functional groups (ammonium and phosphonate): the phosphonate moiety coordinated undercoordinated Pb^2+^ to suppress ion migration, while the ammonium group bonded to halide vacancies to block ion migration channels. This dual-action significantly reduced interfacial trap-state density without compromising the 3D perovskite lattice integrity. Critically, the modification layer concurrently blocked penetration of external species (e.g., oxygen and moisture) and inhibited egress of volatile organic components during thermal stress. Consequently, the FABP-treated perovskite films and devices exhibited enhanced stability against multiple stimuli, including moisture, oxygen, and thermal stress (Fig. 17b) [123]. The interface bridging strategy (IBS) employed A–D–A molecular architectures (exemplified by PMI–F–PMI) to establish robust chemical linkages and realize precise energy-level alignment at the perovskite/fullerene heterointerface (Fig. 17c, d). This approach significantly reduced electron extraction barriers and enhanced interfacial orbital coupling effects. As a result, laboratory-scale cells attained a PCE of 24.62%, while setting a record-high PCE of 18.73% for fullerene-based devices in 156 × 156 mm^2^ modules (Fig. 17e) [148]. Beyond the introduction of modification layers at the perovskite/ETL interface, rational molecular engineering of the fullerene-based electron transport material itself offers a fundamental alternative. For instance, a recently developed phosphate-functionalized fullerene derivative (FuPE), when blended with PCBM, effectively suppresses its self-aggregation. This leads to concomitant enhancements in film uniformity, electron mobility, and interfacial defect passivation capability. Employing this optimized ETL, inverted perovskite solar cells (i-PSCs) have achieved a power conversion efficiency exceeding 26% along with substantially improved operational stability [221]. Similarly, non-fullerene acceptors (NFAs) such as Y6-BO and Y7-BO were employed to strengthen the perovskite/PCBM interface. Owing to the optimal energy-level alignment afforded by Y7-BO and its superior defect passivation capability relative to Y6-BO, electron transfer efficiency across this interface was significantly enhanced (Fig. 17f-h). Notably, Y7-BO-modified inverted perovskite solar modules (i-PSMs) with an aperture area of 1160 cm^2^ recorded a certified PCE of 21.10% (Fig. 17i). Moreover, devices with Y7-BO-stabilized interfaces exhibited exceptional operational stability: encapsulated i-PSCs and i-PSMs retained 90% of their initial PCE after 1400 h under 40% relative humidity (RH), and maintained 94.40% of initial efficiency following 1522 h of continuous MPPT tracking [151]. Furthermore, Guo et al*.* implemented a vapor-deposition-enabled surface reconstruction at the top interface of FAPbI_3_ films, generating a zero-dimensional coordination architecture of Tpy_2_PbI_6_. This modification strategy effectively blocked migration pathways of iodide ions toward the charge transport layer while concurrently suppressing surface ionic defect concentrations. Consequently, industrial-scale PSMs with an aperture area of 785 cm^2^ attained a PCE of 19.60%. Remarkably, after 45 days of real-world outdoor operation, these PSMs demonstrated stability comparable to that of commercial monocrystalline silicon solar cells [155]. Taken together, these surface engineering paradigms—through molecular-scale defect healing, optimized carrier dynamics, and diffusion barrier construction—provide transformative solutions for overcoming the tripartite challenges of ion migration, interfacial non-radiative recombination, and environmental degradation, ultimately advancing perovskite photovoltaic industrialization.

Recent breakthroughs in perovskite photovoltaics underscore the pivotal role of scalable deposition techniques integrated with advanced interface engineering for achieving high-efficiency large-area modules. The development of a formamidinium (FA^+^) supplementation strategy induced secondary grain growth in slot-die-coated MA-free perovskite films, yielding FA-rich compositions with balanced stoichiometry and enlarged grains. This approach optimized interfacial energy-level alignment and enhanced the short-circuit current density, enabling a printed PSMs (active area: 60.84 cm^2^) to achieve a PCE of 17.56% [124]. Notably, post-deposition washing with isopropanol (IPA) facilitated selective removal of methylammonium derivatives (e.g., MACl, MAAc) and triggered beneficial PbI_2_ passivation at grain boundaries via reconstructing lead-rich interfaces, thereby effectively suppressing non-radiative recombination. This dual mechanism empowered rigid n–i–p MAPbI_3_ devices to reach a PCE of 19.91%, while flexible counterparts attaining 17.40%, alongside remarkable operational stability [125]. Further advances in passivation utilize scalable secondary slot-die deposition of benzylammonium iodide (BAI). This treatment enlarged grain sizes, extended photoluminescence lifetimes, and improved spatial homogeneity, thereby boosting device efficiency by 8.50% (20.30% vs. 18.70% control) [135]. In a parallel approach, Zhu et al*.* likewise employed slot-die coating to deposit the passivator 2-chloro-5-(trifluoromethyl)phenylammonium bromide (CF_3_-Cl-PhABr), producing high-quality film morphology with significantly suppressed interfacial recombination [136].

However, translating these remarkable laboratory achievements into commercially viable products remains fraught with significant challenges. Future research must urgently shift from “single-parameter performance optimization” to “comprehensive feasibility assessment” First, it is imperative to develop interfacial materials with broad applicability, low cost, and simple synthesis, ensuring compatibility with diverse perovskite compositions and industrial-scale coating processes such as slot-die coating. Second, it is essential to establish more stringent and comprehensive stability testing protocols to deeply investigate the evolution and failure mechanisms of interfacial structures under multifactorial stress conditions. Finally, a systematic evaluation of the potential impacts of the interfacial modification layers—on thermal management, electrode contact, and long-term carrier transport reliability—must be conducted. Only through deep industry–academia–research collaboration and synergistic innovation in molecular design, process engineering, and stability research can the interfacial “bottleneck” be thoroughly overcome, thereby accelerating the transformation of perovskite photovoltaic technology from a “laboratory champion” to an “industrial titan.”

**Fig. 18:**
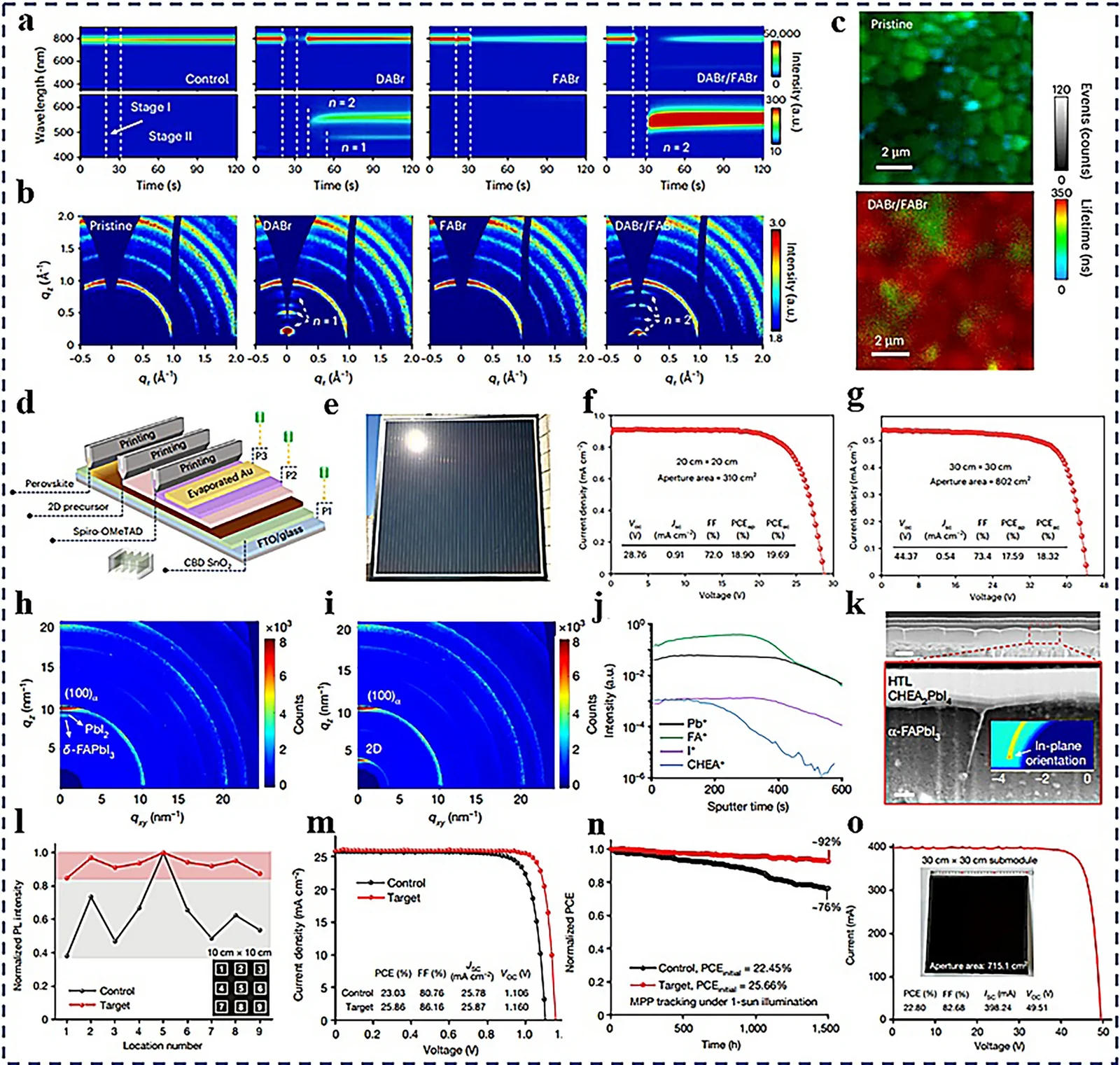
**a** In situ PL measurements of perovskite films with different organic salts post-treatment. **b** 2D GIWAXS data of perovskite films post-treated with different salts. **c** Time-resolved confocal PL mappings. **d** Schematic illustration for the scalable fabrication of large-sized PSMs. **e** Photograph of a 30 cm × 30 cm PSM. **f** *J–V* curve of the champion 20 cm × 20 cm submodule. **g** *J–V* curve of the champion 30 cm × 30 cm small module. Reproduced with permission from Ref. [147]. Copyright 2024, Springer Nature. **h** and **i** GIWAXS patterns of control and target films. **j** Depth profiles of ions in target film, measured by TOF–SIMS. **k** Cross-sectional HR-STEM images of a target PSC sample. **l** PL mapping results at the boundary between the control and target areas, Scale bar, 10 μm. **m** Schematic of CHEAI-induced formation of energy-level alignment and the charge carrier transport process. **n** Normalized PL intensity of 10 × 10 cm^2^ perovskite films. **o** *I–V* curve of the champion 30 × 30 cm^2^ target PSM and photograph (inset) of a 30 × 30 cm^2^ PSM. Reproduced with permission from Ref. [27]. Copyright 2024, Springer Nature

By enabling more uniform defect passivation and simultaneously converting non-photoactive phases (e.g., PbI_2_ and yellow phase δ-FAPbI_3_) into photoactive perovskite compounds, top interface 2D engineering has emerged as a pivotal strategy to bridge the gap between laboratory-scale solar cells and the industrial fabrication of high-performance perovskite solar modules. For instance, Bu et al*.* selectively introduced a system containing formamidinium bromide (FABr) and dodecylammonium bromide (DABr) during post-treatment of 3D formamidinium-cesium perovskite films. This approach drove a transition from mixed-phase (n = 1 and n = 2) to phase-pure (n = 2) 2D perovskites at the surface (Fig. 18a, b). Consequently, a stable 3D/2D heterostructure was formed, exhibiting a more homogeneous morphology, reduced defects (Fig. 18c), and faster interfacial charge transport. Significantly, this passivation strategy was compatible with printing techniques. Fully slot-die-coated large-area modules with aperture areas of 310 and 802 cm^2^ achieved optimal stabilized efficiencies of 18.90% and 17.59%, respectively, demonstrating the feasibility of scalable manufacturing (Fig. 18d-g) [147]. Building upon the pursuit of homogeneous and phase-selective passivation, Zhao et al*.* developed an effective impurity-healing strategy utilizing a carbazole-based halide-amine intercalator (CHEAI). This method converted excess PbI_2_ and residual light-absorbing δ-FAPbI_3_ impurities at the surfaces and grain boundaries of 3D FAPbI_3_ perovskite into 2D perovskite (Fig. 18h-k), enabling uniform passivation (Fig. 18l). Simultaneously, it constructed efficient pathways for charge carrier transport and extraction from the 3D perovskite bulk to the HTL (Fig. 18m). Consequently, FAPbI_3_-based small-area (0.085 cm^2^) PSCs achieved the highest PCE of 25.86% with enhanced operational stability. More importantly, demonstrating the compelling scalability of this approach, a certified PCE of 22.80% with an impressive fill factor (*FF*) of 82.68% was attained on PSMs with an aperture area of 715.1 cm^2^ (Fig. 18n, o) [27]. This result showcases the effectiveness of impurity-healing interfacial engineering for scalable fabrication of high-performance PSMs with minimal efficiency loss. Subsequently, Zhao’s group developed a solution–vacuum hybrid batch fabrication technology. This approach allowed precise deposition of nanoscale two-dimensional capping layers via all-vacuum evaporation on solution-processed three-dimensional (3D) bulk perovskite films. The composition and stoichiometry of these entirely vacuum-deposited 2D perovskite capping layers were precisely modulated, thereby effectively passivating surface defects and healing intrinsic pinholes inherent to solution-processed films. Based on this strategy, they successfully fabricated large-area (30 cm × 30 cm) pinhole-free perovskite submodules, achieving the highest PCE of 22.10% with a certified value of 21.79%. This breakthrough effectively integrates the rapid processing advantages of solution techniques with the precise control of vacuum deposition, paving a novel and promising technological pathway for efficient and reproducible large-scale production of perovskite photovoltaic modules [157].

#### Ion Diffusion Barrier Engineering

Beyond chemical modification aimed at surface defect passivation and energy-level alignment, constructing effective physical barriers against ion diffusion at critical internal locations within the device—such as the perovskite/charge transport layer interface or the laser-scribed P2/P3 regions—represents a core strategy for suppressing halide ion migration and enhancing the series connection stability and long-term reliability of perovskite modules. Such barrier layers are typically realized by introducing thin-film materials with dense microstructures or specific chemical functionalities.

**Fig. 19:**
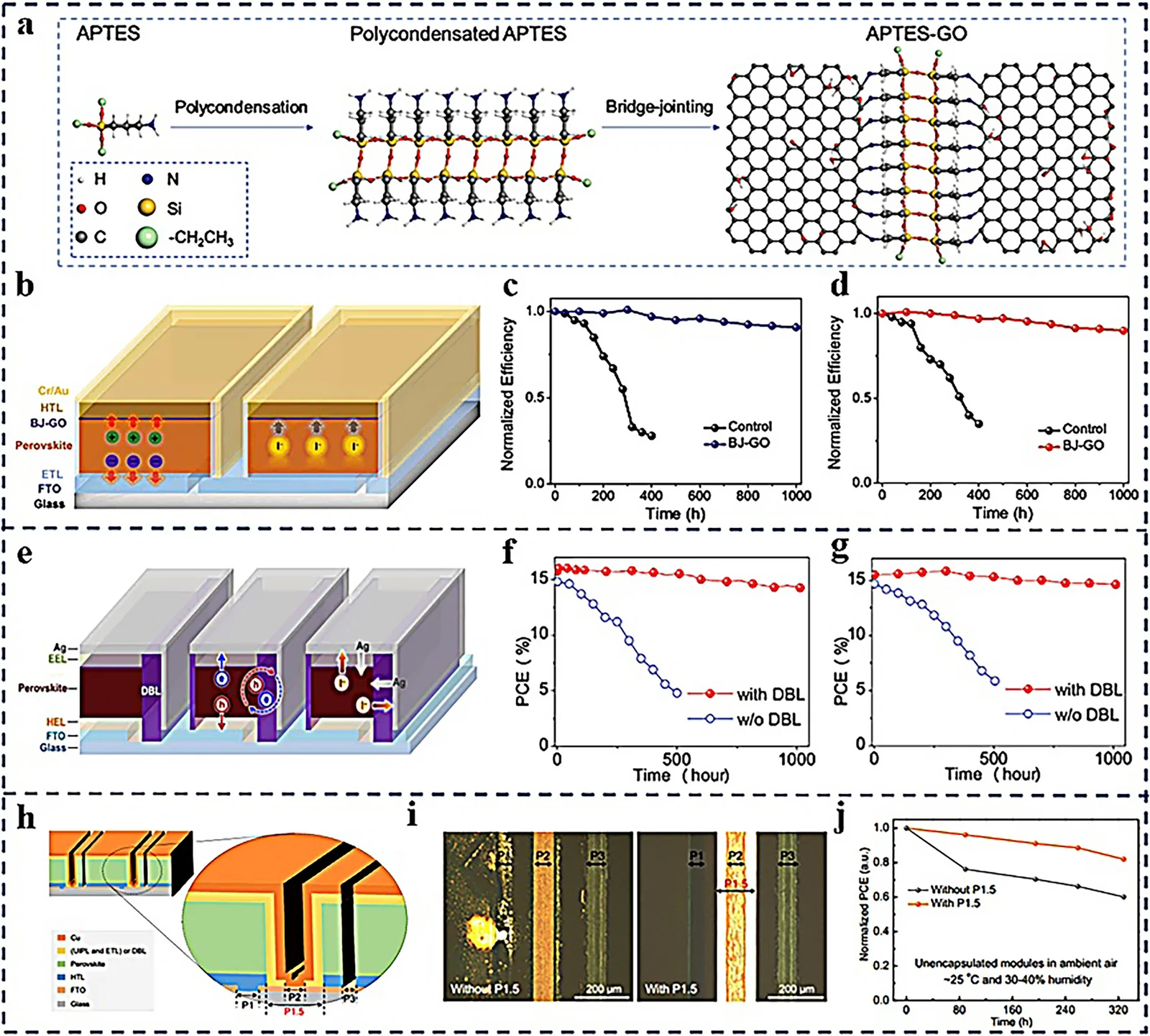
**a** Schematic illustration of the formation process of APTES-GO. **b** Schematic diagram of the structure of PSMs. **c** Thermal stability of the encapsulated module with or without BJ-GO. **d** Stability of the encapsulated module with or without BJ-GO at the MPPT. Reproduced with permission from Ref. [142]. Copyright 2021, John Wiley and Sons. **e** Illustration interfacial charge transfer and recombination in PSMs. **f** and** g** In heating aging test at 85 °C with relative humidity of about 85% for 1,000 h, and under a UV-filtered simulated sunlight at 60 °C in ambient air and maintained at the MPPT. Reproduced with permission from Ref. [141]. Copyright 2019, Cell Press. **h** Schematic diagram of the structure of a module with P1.5 scribing. **i** Optical micrograph of the interconnect region of the modules after aging. **j** Aging stability tracking of modules in ambient air (25 °C, 30% ~ 40% humidity). Reproduced with permission from Ref. [152]. Copyright 2024, Springer Nature

The intrinsic structural fragility of perovskites, exacerbated by the diffusion of ions (e.g., Pb^2+^ and I^−^, with I^−^ being the most mobile species) that triggers irreversible degradation pathways, constitutes a fundamental barrier to realizing operationally stable perovskite devices [222]. To further suppress iodide ion migration, Han et al*.* developed a chemical suturing technique for fabricating a large-area, ultrathin layer of bridged graphene oxide (BJ-GO) (Fig. 19a). This interlayer was precisely positioned between the perovskite absorber and the HTL (Fig. 19b), yielding a triple synergistic effect: it physically blocked iodide ion diffusion toward the HTL, passivated undercoordinated lead defects through multidentate coordination by C = O groups, and induced highly ordered self-assembly of the HTL via surface energy modulation. Consequently, carrier mobility was enhanced to 2.29 × 10^–2^ cm^2^ V^−1^ s^−1^, marking a nearly tenfold improvement. Leveraging this strategy, a large-area module (aperture area: 35.80 cm^2^) exhibited a certified PCE of 15.30% with negligible hysteresis. Furthermore, the device exhibited exceptional stability, retaining > 91% of its initial PCE after 1000 h of aging under 85 °C and 85% relative humidity (85 °C/85% RH) and maintaining > 90% of its initial efficiency under continuous MPPT at 60 °C under 1-sun equivalent illumination (Fig. 19c, d) [142]. As a complementary strategy, Han and co-workers employed a low-temperature solution process to construct a two-dimensional diffusion barrier layer (g-C_3_N_4_) at the top interface of the perovskite film and the P2-laser-scribed regions (Fig. 19e). This approach suppressed the iodide ion diffusion rate by 10^3^–10^7^-fold, significantly enhancing the operational stability of perovskite solar modules. Consequently, the fabricated 36 cm^2^ module achieved a certified power PCE of 15.60%, retaining > 95% of its initial PCE after 1000 h of thermal aging at 85 °C and maintaining > 91% of the initial PCE after 1000 h of continuous operation under AM 1.5G illumination (Fig. 19f, g) [141]. More intriguingly, You et al*.* creatively devised a straightforward one-step P1.5 scribing process, which enabled the in situ formation of an effective diffusion barrier layer (Fig. 19h, i). This design thereby significantly alleviated the degradation issues triggered by iodine ion migration. Consequently, the fabricated devices retained over 90% of their initial photoelectric conversion efficiency after 1000 h of continuous operation under MPPT tracking (Fig. 19j) [152].

## Photoluminescence Characterization: An in situ Diagnostic Tool for Process Optimization

As highlighted throughout this review, in situ and real-time PL characterization techniques—including steady-state PL imaging, PL spectroscopy, and time-resolved PL mapping—provide a unique window into the complex dynamics of slot-die coating. However, translating these optical signatures into specific process adjustment directives is the crucial bridge connecting fundamental research with industrial optimization. Based on current research, we can establish a preliminary “PL Signature–Process Issue” diagnostic framework (Table 3). This framework aims to help process engineers rapidly correlate observed non-ideal PL signals with potential thin-film formation or interface problems and point toward possible optimization directions. Establishing and continually refining such diagnostic matrices is pivotal for transitioning slot-die process optimization from empirical “trial-and-error” to a science-driven, physics-informed intelligent control paradigm. Looking ahead, the integration of machine learning algorithms for automated pattern recognition of high-throughput PL imaging and spectral datasets holds great promise for enabling real-time anomaly detection and self-corrective process adjustments. This synergy between advanced optical diagnostics and adaptive control will ultimately pave the way for the robust, high-yield manufacturing of photovoltaic-grade perovskite films with exceptional uniformity and performance.

**Table 3:** Diagnostic matrix of common PL features for slot-die-coated perovskite films

Observed PL feature	Potential process/material issue indicated	Physical origin and diagnosis	Suggested optimization direction
**Spatially Inhomogeneous PL Imaging**	**1. Non-uniform drying kinetics:** Uneven gas quenching flow/temperature distribution, or localized low-pressure zones in vacuum-assisted drying. **2. Substrate temperature gradient:** Non-uniform thermal field from hotplate or NIR source. **3. Unstable ink delivery:** Pump fluctuation or partial clogging of the slot-die head.	Spatial variations in film thickness, crystallinity, or phase purity, leading to inconsistent carrier generation and recombination efficiency.	1. Optimize drying system design (e.g., use a “comb nozzle” instead of a linear one). 2. Calibrate and homogenize the heat source. 3. Inspect and stabilize the ink delivery system; filter precursor colloids.
**Significantly Lower PL Intensity over Large Area vs. Spin-Coated Reference**	**1. Excessively slow solvent removal: **Leads to overly coarse grains and/or abundant pinholes. **2. Insufficient nucleation density:** Lack of effective quenching (gas, vacuum, anti-solvent) to trigger instantaneous supersaturation. **3. Ambient interference:** High humidity leading to intermediate phase or hydrate formation.	Extremely high density of non-radiative recombination centers (defects) or insufficient film coverage.	1. Introduce or enhance auxiliary drying steps (e.g., vacuum flashing, synchronized N_2_ knife quenching). 2. Modify ink formulation with additives that promote nucleation. 3. Strictly control the coating environment (temperature, humidity).
**Spatial Gradient in TRPL Lifetime**	**1. “Coffee-ring” edge effects: **Solute migration induced by the drying front. **2. Non-uniform annealing temperature: **Causes spatial variation in grain boundary properties or residual stress. **3. Varied interfacial contact:** Non-uniform coverage or energy levels of the underlying layer (e.g., ETL/HTL).	Inconsistent carrier diffusion and recombination kinetics across space, often related to defect distribution or interfacial energy barrier gradients.	1. Optimize ink rheology (e.g., adjust viscosity, surface tension) or employ multistep coating. 2. Improve the uniformity of the annealing process. 3. Ensure high uniformity and full coverage of the underlying functional layers.
**Spatial Drift of PL Peak Position (Color) or Multiple Peaks**	**1. Halide phase segregation:** Ion migration and phase separation occurring in compositionally inhomogeneous regions under light/electrical bias. **2. Residual intermediate phases:** e.g., δ-FAPbI_3_ or PbI_2_ due to incomplete conversion or improper annealing. **3. Thickness gradient:** Causing optical interference effects (requires correlation with absorption spectra).	Inhomogeneity in the stoichiometry or crystal structure of the perovskite lattice at micro- or macroscales.	1. Optimize perovskite composition (e.g., use mixed cations/halides); introduce additives that suppress ion migration. 2. Optimize two-step conversion or annealing conditions to ensure complete reaction. 3. Precisely control coating parameters (gap, speed, flow rate) to guarantee thickness uniformity.
In situ **PL Monitoring Shows Delayed or Overly Rapid Crystallization**	**1. Ink aging or colloidal pre-crystallization:** Poor stability of precursor ink, leading to uncontrolled aggregation before coating. **2. Improper solvent/additive combination:** The kinetic window for intermediate phase formation/decomposition does not match the coating–drying sequence. **3. Incorrect quenching timing:** Initiation of gas or anti-solvent quenching is misaligned with the critical nucleation period.	The crystallization pathway deviates from the intended optimal kinetic trajectory.	1. Use freshly prepared or stabilized ink formulations. 2. Fine-tune the thermodynamic and kinetic stability of intermediate phases through solvent and additive engineering. 3. Establish a quenching trigger logic based on in situ PL feedback for adaptive process control.

Diagnostic Note: This table serves as a general guide. In practice, cross-validation using multiple PL techniques (imaging, spectroscopy, lifetime) is essential, and should be combined with other in-line (e.g., reflectance/transmittance) and ex-situ (e.g., SEM, XRD) characterizations for final confirmation

## Conclusion and Outlook

**Fig. 20:**
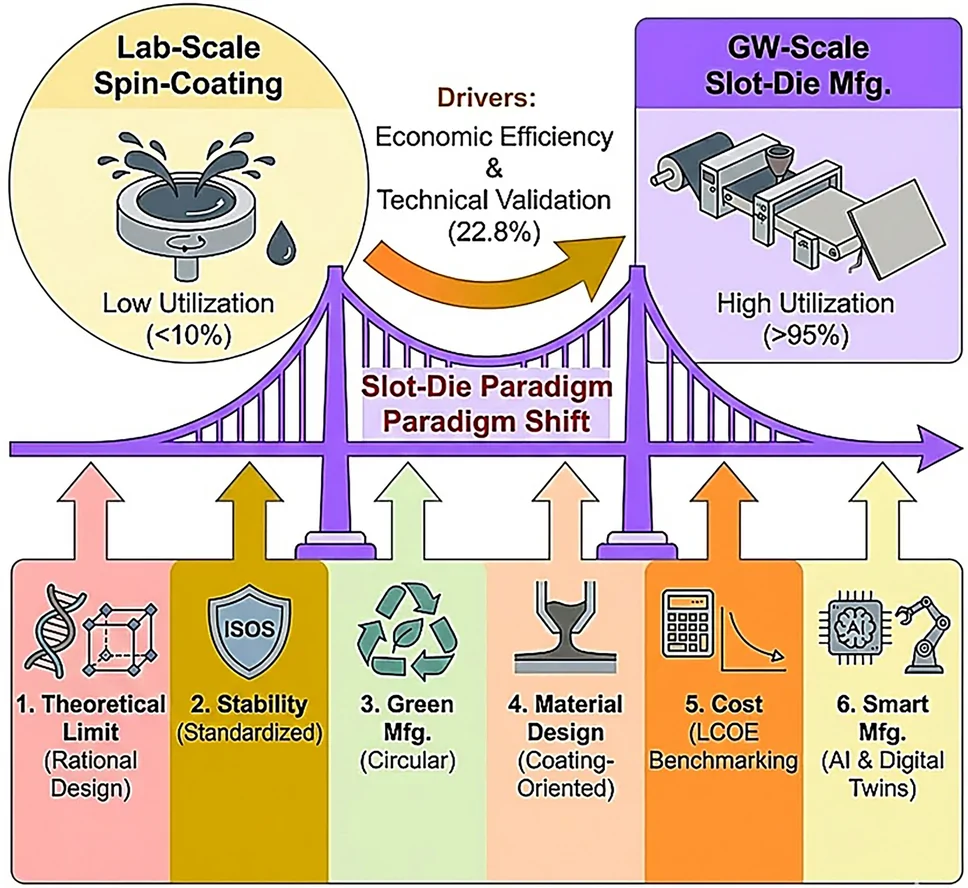
Paradigm shift toward GW-scale perovskite photovoltaics via slot-die coating: Strategic Pillars

Slot-die coating is by no means a simple alternative to spin coating; rather, it represents a critical technological pathway for enabling the gigawatt-scale manufacturing era of perovskite solar cells, as outlined in our strategic roadmap (Fig. 20). The core driver of its industrialization lies in its exceptional economic efficiency: compared to the less than 10% material utilization rate of spin-coating, slot-die coating can achieve a material utilization rate exceeding 95%. This near-zero-waste characteristic directly reduces the unit manufacturing cost of the perovskite active layer—a significant component of photovoltaic module material costs—thereby laying the foundation for lowering both the cost per watt ($/W) and the levelized cost of electricity (LCOE) [223, 224]. This constitutes the fundamental economic rationale for its industrialization. Meanwhile, technical feasibility has been robustly validated. Modules fabricated using this process have achieved an outstanding efficiency of 22.80% [27], fully demonstrating the performance potential of this technological route. However, to translate laboratory success into disruptive industrial competitiveness and propel the technology from pilot lines to gigawatt-scale mass production, we must squarely address and overcome the core challenges in transitioning from “sample fabrication” to “scaled manufacturing”. This requires systematic breakthroughs across the following dimensions:

(1) Approaching the Theoretical Limit: From Empirical Exploration to Rational Design. The efficiency gap fundamentally stems from the multiscale regulation of crystallization dynamics. Future efforts must decouple the nucleation growth kinetics between spin-coating and large-area coating, clarifying whether the physical limits arise from intrinsic thermodynamic boundaries or extrinsic kinetic defects. Breakthrough pathways include: 1) Ink Design Guided by Materials Genomics and Fluid Dynamics: Utilizing high-throughput computation and machine learning to screen multicomponent systems with broad crystallization windows, low formation energies, and high defect tolerance, and optimal rheological properties for stable coating. 2) Field-Assisted Intelligent Crystallization: Developing spatiotemporally ordered field-assisted processes (e.g., optical, thermal, acoustic, magnetic, or electrical) to transition from “stochastic nucleation” to “directed self-assembly” (For instance, developing near-field infrared radiation to modulate the temperature profile, or utilizing ultrasound to guide grain preferential growth.). 3) Cross-Scale Heterointerface Integration: Synergistically combining molecular-level passivation with module-level design to suppress interfacial non-radiative recombination and minimize dead-area losses.

(2) Resolving Stability Challenges: From Passive Protection to Active Healing, Grounded in Standardized Evaluation. Stability must be a “designed-in” attribute, not an afterthought. The foundation of all stability research and claims must be built upon internationally recognized, standardized testing protocols as outlined in the stability assessment Sect. 3.2. Future efforts must focus on: 1) Standardized Testing as a Prerequisite: Adopt and report data under relevant ISOS protocols from the early stages of research. This ensures credibility, comparability, and accelerates reliable technology down-selection. 2) Intrinsic Stabilization via Lattice Engineering: Enhancing inherent robustness via lattice stress engineering, phase distribution control, and self-passivating defects. 3) Smart Encapsulation with Functionality: Developing intelligent encapsulation systems with in situ monitoring and self-healing functionalities (e.g., microcapsule-based healing agents, dynamic reversible cross-linking polymers). 4) Lifetime Prediction Based on Reliable Data: Constructing power-plant-level reliability management and lifetime prediction platforms based on digital twins (virtual replicas of physical systems) and predictive maintenance. All models and predictions must be calibrated using reliable data generated from standardized testing.

(3) Green Manufacturing and Circular Economy: Defining Sustainable Standards for Next-Generation Photovoltaics. Solvent toxicity remains a “one-vote veto” barrier to industrial adoption. Future solvent innovation must transcend mere “substitution” and establish a full life cycle greenness evaluation system: 1) Bio-Based Green Solvents: Designing novel solvent systems with strong coordination capability, biodegradability, and recyclability. 2) Precision-Programmed Evaporation Pathways: Integrating artificial intelligence and fluid dynamics simulations to achieve intelligent control from meniscus flow fields to drying kinetics, yielding defect-free films. 3) From Green Processes to Circular Design: Promoting integrated technological solutions for low-carbon module fabrication and end-of-life material recycling.

(4) Material Design for Coating-Oriented Manufacturing: From Laboratory Formulations to Industrial Inks. The current paradigm for perovskite materials is largely based on spin-coating, which operates under extreme, non-equilibrium conditions. This results in ink formulations optimized for a narrow kinetic pathway that is misaligned with the slower, evaporation-controlled regime of slot-die coating. A dedicated research thrust on “coating-oriented material design” is therefore essential. Key directions include: 1) Rheology-engineered inks with tailored viscoelastic properties to stabilize the coating meniscus and ensure uniform deposition. 2) Ink components that provide a wide “coatable zone” to desensitize film formation to ambient fluctuations. 3) Formulations yielding perovskite films with intrinsic resistance to thermal, atmospheric, and mechanical stresses encountered in in-line production. The ultimate goal is to shift from adapting laboratory recipes to intentionally designing materials for the slot-die coating environment, unlocking its full potential for yield, speed, and consistency.

(5) Manufacturing Cost and Competitiveness: From Qualitative Advantages to Quantitative Analysis. A comprehensive assessment of its industrial competitiveness requires systematic techno-economic analysis (TEA). Future research should not only demonstrate high efficiency but also provide key economic indicators and data: 1) Material Cost: Compare the perovskite ink utilization of slot-die coating (> 95%) versus spin coating (< 10%), quantifying the cost savings per unit area of the active layer. 2) Manufacturing Cost: Analyze the full production cost structure, including capital expenditure (coating heads, drying systems, R2R production lines), energy consumption (heating, vacuum, gas usage), facility overhead, and labor. 3) Throughput and Output: Evaluate coating line speed (m min^–1^), production capacity per unit time (m^2^ h^–1^), and the associated depreciation cost allocation of equipment. 4) Yield and Quality Cost: Explore the trade-off between initial investment and the levelized cost of electricity (LCOE) advantages brought by high yield and long-term operational stability. Establishing open and transparent TEA models and benchmarking them against mature technologies such as crystallinie silicon and thin-film photovoltaics are essential for attracting industrial investment and defining R&D priorities. Only by demonstrating unambiguous competitiveness in LCOE—on the foundation of achieving target efficiency and stability—can slot-die-coated perovskite photovoltaics truly achieve commercial disruption.

(6) Reinventing Manufacturing Paradigms: Building “Lighthouse Factories” for Perovskite Photovoltaics. The core of future competitiveness lies in smart manufacturing capabilities. The fourth-generation photovoltaic production line must be driven by a closed-loop “data–algorithm–process” system: 1) Digital Twin Systems: Establishing cross-scale virtual mappings from molecular simulations to production line operations, enabling virtual iteration and optimization of process parameters. 2) In-Line Full Inspection and AI Decision-Making: Integrating advanced sensing (e.g., terahertz imaging, spectral tomography) with reinforcement learning algorithms for real-time quality assessment and adaptive control. 3) All-Slot-Die-Coating Process Integration: The development of fully integrated, continuous production lines based entirely on slot-die coating—depositing electron transport layers, perovskite absorbers, hole transport layers, and even electrode or encapsulation precursors—is a key strategic pathway to low-cost industrialization.

In essence, the transition from spin coating to slot-die coating is far more than a change in tools; it is a fundamental shift in the scientific and engineering paradigm for perovskite photovoltaics. Success hinges on co-advancing the targets of efficiency, sustainability, and stability with the critical enablers of smart manufacturing and purpose-built materials. The journey from laboratory-scale champions to industrial titans is challenging, but a systematic research agenda focused on these interconnected pillars, grounded in standardized evaluation and a coating-centric design philosophy, will accelerate the realization of perovskite solar cells as a cornerstone of the global renewable energy landscape. We call for deep, interdisciplinary collaboration across academia, national laboratories, and industry to jointly propel this transformative technology from promising blueprint to global energy reality.
